# Activation of GPR40 produces mechanical antiallodynia via the spinal glial interleukin-10/β-endorphin pathway

**DOI:** 10.1186/s12974-019-1457-9

**Published:** 2019-04-13

**Authors:** Xiao-Fang Mao, Hai-Yun Wu, Xue-Qi Tang, Usman Ali, Hao Liu, Yong-Xiang Wang

**Affiliations:** King’s Lab, Shanghai Jiao Tong University School of Pharmacy, 800 Dongchuan Road, Shanghai, 200240 China

**Keywords:** GPR40, β-Endorphin, IL-10, Spinal cord, Microglia, Astrocytes

## Abstract

**Background:**

The G protein-coupled receptor 40 (GPR40), broadly expressed in various tissues such as the spinal cord, exerts multiple physiological functions including pain regulation. This study aimed to elucidate the mechanisms underlying GPR40 activation-induced antinociception in neuropathic pain, particularly related to the spinal glial expression of IL-10 and subsequent β-endorphin.

**Methods:**

Spinal nerve ligation-induced neuropathic pain model was used in this study. β-Endorphin and IL-10 levels were measured in the spinal cord and cultured primary microglia, astrocytes, and neurons. Double immunofluorescence staining of β-endorphin with glial and neuronal cellular biomarkers was also detected in the spinal cord and cultured primary microglia, astrocytes, and neurons.

**Results:**

GPR40 was expressed on microglia, astrocytes, and neurons in the spinal cords and upregulated by spinal nerve ligation. Intrathecal injection of the GPR40 agonist GW9508 dose-dependently attenuated mechanical allodynia and thermal hyperalgesia in neuropathic rats, with *E*_max_ values of 80% and 100% MPE and ED_50_ values of 6.7 and 5.4 μg, respectively. Its mechanical antiallodynia was blocked by the selective GPR40 antagonist GW1100 but not GPR120 antagonist AH7614. Intrathecal GW9508 significantly enhanced IL-10 and β-endorphin immunostaining in spinal microglia and astrocytes but not in neurons. GW9508 also markedly stimulated gene and protein expression of IL-10 and β-endorphin in cultured primary spinal microglia and astrocytes but not in neurons, originated from 1-day-old neonatal rats. The IL-10 antibody inhibited GW9508-stimulated gene expression of the β-endorphin precursor proopiomelanocortin (POMC) but not IL-10, whereas the β-endorphin antibody did not affect GW9508-stimulated IL-10 or POMC gene expression. GW9508 increased phosphorylation of mitogen-activated protein kinases (MAPKs) including p38, extracellular signal-regulated kinase (ERK), and c-Jun N-terminal kinase (JNK), and its stimulatory effects on IL-10 and POMC expression were blocked by each MAPK isoform inhibitor. Spinal GW9508-induced mechanical antiallodynia was completely blocked by intrathecal minocycline, IL-10 neutralizing antibody, β-endorphin antiserum, and μ-opioid receptor-preferred antagonist naloxone.

**Conclusions:**

Our results illustrate that GPR40 activation produces antinociception via the spinal glial IL-10/β-endorphin antinociceptive pathway.

**Electronic supplementary material:**

The online version of this article (10.1186/s12974-019-1457-9) contains supplementary material, which is available to authorized users.

## Introduction

The orphan G protein-coupled receptor 40 (GPR40), also known as free fatty acid (FFA) receptor 1, was first discovered in 1997 [1] and deorphanized in 2003 [2]. Belonging to the family A of the G protein-coupled receptor, it is activated by saturated/unsaturated medium- and long-chain FFA [2, 3]. GPR40 is widely expressed in various tissues, such as the brain, pancreas islet, heart, and spinal cord, with preferential expression in pancreatic β-cells [2–5]. GPR40 activation exerts multiple physiological functions in glucose metabolism, including regulation of the secretion of insulin [3], incretin [6], and glucagon [7]. Accumulative studies have proved GPR40 to be a promising target molecule for the treatment of type 2 diabetes mellitus, and its ligands are under clinical investigations as antidiabetic drugs [8–11]. In addition, GPR40 produces anti-neuroinflammation [12] and neuroprotection [13, 14]. GPR40 activation directly led to ERK activation, which subsequently performed profound functions in neuronal plasticity and long-term memory [15, 16]. The GPR40 agonist GW9508 significantly improved amyloid β-impaired cognitive performance in a mouse model of Alzheimer’s disease [17].

GPR40 has been extensively demonstrated to be implicated with pain regulation. The GPR40 endogenous agonist docosahexaenoic acid (DHA) or exogenous agonist GW9508, given orally, intraperitoneally, or intracerebroventricularly, remarkably reduced pain behaviors in the mouse models of inflammatory pain or visceral pain induced by acetic acid, formalin, complete Freund’s adjuvant (CFA), carrageenan, or cyclophosphamide [14, 18–22]. Given intracerebroventricularly, they also significantly attenuated mechanical hyperalgesia induced by bilateral carotid artery occlusion in the mouse model of central post-stroke pain, which was reversed by the GPR40 antagonist GW1100 [23]. Furthermore, intrathecal injection of GW9508, DHA, and MEDICA16 significantly reduced mechanical allodynia and thermal hyperalgesia in the mouse models of neuropathic pain induced by spinal nerve injury and inflammatory pain induced by CFA and carrageenan [24], although a study reported these agents, given intrathecally, were ineffective in reducing formalin-induced pain [18].

The mechanisms underlying GPR40 activation-induced antinociception remain unclear. It was reported that the descending inhibitory serotonergic and noradrenergic systems contributed to supraspinal GPR40 antinociception since intracerebroventricular injection of GW9508 facilitated the release of noradrenaline and 5-HT in the spinal cord and that the serotonergic and noradrenergic depleting and blocking agents 6-hydroxydopamine, dl-*p*-chlorophenylalanine, yohimbine, and WAY100635 inhibited GW9508-induced antinociception in the mouse formalin test [25]. DHA-induced antinociception also possibly occurred through the inhibition of microglia and astrocyte activation-induced spinal neuroinflammation and the secretion of inflammatory cytokines [23, 26]. On the other hand, the secretion of β-endorphin has been assumed to be associated with GPR40 activation-induced antinociception, as the GPR40 agonists accelerated β-endorphin release from the hypothalamus, and pretreatment with the β-endorphin antiserum, opioid receptor antagonist naloxone, and μ-opioid receptor antagonists β-funaltrexamine and CTOP blocked DHA- and GW9508-induced antinociception in the mouse inflammatory pain models of acetic acid, formalin, and CFA [18, 20, 21, 27, 28].

We recently uncovered that various glucagon-like peptide-1 (GLP-1) receptor agonists (exenatide, WB4–24, and shanzhiside methylester), Cynanchi-derived cynandione A, and Aconitum-derived bulleyaconitine A, bullatine A, and lappaconitine effectively relieved chronic pain through the microglial secretion of β-endorphin and dynorphin A [29–35]. In addition, interleukin-10 (IL-10) is a well-known anti-inflammatory and immunosuppressive cytokine, expressed in astrocytes [36, 37] and microglia [38, 39], and its antinociception has been markedly confirmed in various pain models [40–45]. Intrathecal injection of exogenous IL-10-induced mechanical antiallodynia was mediated by the spinal microglial expression of β-endorphin [39]. Moreover, the GLP-1 receptor agonist exenatide stimulated the spinal expression of β-endorphin and produced mechanical antiallodynia in neuropathic pain through the autocrine microglial secretion of IL-10, via a cAMP/PKA/p38β/CREB signal pathway [46]. Thus, it is possible that GPR40 activation-induced β-endorphin secretion, likewise GLP-1 receptor activation, is mediated by IL-10 expression.

The present study aimed to investigate the mechanisms underlying GPR40 activation-induced antinociception in neuropathic pain, especially related to the spinal glial IL-10/β-endorphin antinociceptive pathway. We first tested the effect of GW9508, given intrathecally, on mechanical allodynia and thermal hyperalgesia in neuropathic rats. Furthermore, we studied the glial expression of IL-10 and β-endorphin in the spinal cord and cultured primary cells and the signal transduction pathways. Lastly, we explored the association between the spinal glial expression of IL-10/β-endorphin and GW9508-induced mechanical antiallodynia. Our study, for the first time, reveals that GPR40 activation produces antinociception in neuropathic pain through the spinal glial expression of IL-10 and subsequent β-endorphin, highlighting the role of the newly discovered glial IL-10/β-endorphin pathway in pain transmission and transduction.

## Materials and methods

### Drugs and reagents

GW9508, naloxone, GW1100, and AH7614 were purchased from Target Mol (Shanghai, China), Sigma-Aldrich (St. Louis, MO, USA), Cayman Chemical Company (Ann Arbor, Michigan, USA), and ApexBio (Houston, TX, USA), respectively. SP600125, SB203580, and U0126 were from Selleck Chemicals (Houston, TX, USA). Polyclonal antiserum against β-endorphin was purchased from Abcam (Cambridge, UK), and polyclonal antibodies against IL-10 and β-endorphin were obtained from Thermo Fisher Scientific (Vienna, Austria) and Phoenix Pharmaceuticals, Inc. (Burlingame, CA, USA), respectively. GW1100 or AH7614 was dissolved in 30% dimethyl sulfoxide (DMSO) and 70% polyethylene glycol (PEG) 400 or 50% PEG 400 in 0.9% normal saline. All other drugs or reagents were dissolved or diluted in normal saline.

### Experimental animals

Male or female 1-day-old neonatal and male adult (160–180 g) Wistar rats were from the Shanghai Experimental Animal Institute for Biological Sciences (Shanghai, China). The adult rats were kept in the Laboratory Animal Center of Shanghai Jiao Tong University in a temperature- and humidity-controlled environment with an automatic 12-h light/dark cycle and access to food and water ad libitum. The animals were under 3–5 days’ adaptation before being used for any experiments. All the research protocols were approved by the Animal Care and Welfare Committee of the Shanghai Jiao Tong University (Shanghai, China) and carried out in accordance with the animal care guidelines of the US National Institutes of Health.

### Primary cell culture

Primary microglia, astrocytes, and neurons were isolated from the spinal cords of 1-day-old neonatal rats. The isolated spinal cords were minced and incubated with 0.05% trypsin-EDTA (Invitrogen, Carlsbad, CA, USA) for 7 min. The dissociated cells were suspended in DMEM supplemented with 10% (*v*/*v*) fetal bovine serum, penicillin (100 U/ml), and streptomycin (100 μg/ml). The cell suspension was filtered by a 40-μm cell strainer and then plated into 75-cm^2^ tissue cultural flasks (1 × 10^7^ cells/flask) pre-coated with poly-l-lysine (0.1 mg/ml) and maintained at 37 °C in a 5% carbon dioxide incubator. For microglial cell preparation, the aliquots were collected after shaking the flasks at 260 rpm, 37 °C for 1.5–2 h after 10 days of culture. The cell suspension was then transferred to new plates, and unattached cells were removed by washing with phosphate-buffered saline (PBS) before drug treatments. Harvested microglial cells exhibited a purity > 95%, as determined by the microglial cellular marker ionized calcium binding adaptor molecule-1 (Iba-1) immunoreactivity [29, 39].

For the astrocyte preparation, the mixed cells were incubated with 4 ml of 0.05% trypsin-EDTA in the cell incubator for 3 min to separate the oligodendrocytes. After neutralization, the floating cell suspension was discarded. The remaining monolayer of astrocytes was then trypsinized and sub-cultured in new plates for further experiments. Harvested astrocytes exhibited a purity > 90%, as determined by the astrocytic cellular marker glial fibrillary acid protein (GFAP) immunoreactivity [29].

For neuronal cells, the cell suspension was plated into a 10-cm dish after being filtered by 40 μm for about 30 min to remove non-neural cells, and then, the cell suspension was plated into poly-l-lysine pre-coated plates. After incubation with complete DMEM for 1–2 h, the medium was changed to the Neurobasal (Invitrogen) containing 2% B27 supplement and 0.5 mM glutamine. The following experiments were performed 3–4 days later. Harvested neurons exhibited a purity of 85%, as determined by the neuronal cellular marker neuronal specific nuclear protein (NeuN) immunoreactivity [29].

### Intrathecal catheterization and injection in rats

An 18-cm PE-10 catheter (OD 0.55 mm, ID 0.30 mm; AniLab Software & Instruments Co., Ningbo, China) was inserted into the lumbar level of the spinal cord under inhaled isoflurane anesthesia (4% for induction and 2% for maintenance) run by a gas anesthesia system (Ugo Basile, Comerio, Italy). The intrathecal catheterization was checked after 1-week recovery from surgery by intrathecal administration of 10 μl 4% lidocaine with 15 μl normal saline flush. Intrathecally catheterized rats that had no motor impairments after surgery and developed instant bilateral hindlimb paralysis after lidocaine injection were included in further research. For intrathecal injection, 10 μl of the drug solution was administered in a 50-μl syringe (Shanghai Anting Micro-Injector Factory, Shanghai, China) followed by a 15-μl saline flush.

### Rat model of neuropathic pain and behavior assessment

After environmental adaption, L5/L6 spinal nerve ligation surgery was performed on rats under inhaled isoflurane anesthesia as described previously [47, 48]. Briefly, the left L5 and L6 spinal nerves were isolated and tightly ligated with 6–0 silk thread. After ligation, the wound was sutured after giving antibiotic and the rats were allowed to recover for about 2 weeks. As intrathecal injection was needed in the research, intrathecal catheterization was performed at the same time just before nerve ligation. After recovery, only rats with significant unilateral allodynia to mechanical stimulation (hindlimb withdrawal thresholds in the operated side < 8 g) and with no motor deficits were included in the further experiments.

To evaluate mechanical allodynia, the animals were acclimatized to transparent plexiglass boxes on a metal grid for about 30 min. The hindlimb withdrawal threshold was measured by using a 2450 CE Electronic Von Frey hair (IITC Life Science Inc., Woodland Hill, CA, USA). The electronic handheld transducer with a no. 15 monofilament (with forces ranging from 0.1 to 90 g) was applied perpendicularly to the base of the third and fourth toe of the hindpaws with increasing force until a sudden withdrawal. The lowest force to induce the withdrawal response was considered as the threshold. The average of three repeated measurements at a 2-min interval was recorded as each hindlimb for each time point.

To assess thermal hyperalgesia, the rats were placed in plexiglass boxes on a heated glass surface (about 37 °C) for about 30 min for adaption. After that, a radiant heat source (setting at a low density of 50) was applied to the plantar medial surface of each hindpaw. The hindpaw withdrawal latency was measured by a 390G Plantar Test Analgesia Meter (IITC Life Science Inc.). The paw withdrawal latency was defined as the time from the onset of radiant heat application to the withdrawal of the hindpaw. The cutoff latency was set at 30 s to avoid tissue damage. Each value at a time point was the mean value of three repeated measurements at a 2-min interval between tests.

Neuropathic rats, starting the drug testing 2–3 weeks after the spinal nerve ligation, were randomly assigned to experimental groups, and the behavior observations were performed in a manner blind to the experimental conditions.

### RNA extraction and quantitative real-time PCR measurement

For tissue samples, spinal lumbar enlargements (L3–5) from rats were isolated and homogenized using an electronic homogenizer at 4000 rpm for 15 s in TRIzol (Invitrogen) on ice. For cell samples, cells were collected after drug treatment and lysed in TRIzol for 5 min. Total RNAs extracted from the spinal cords and cells were reversely transcribed into cDNA using a ReverTra Ace qPCR RT kit (Toyobo Co., Osaka, Japan) according to the manufacturer’s protocols. Quantitative real-time PCR was then carried out by using the Realmaster Mix (SYBR Green I) (Toyobo Co.) in a Mastercycler ep realplex (Eppendorf, Hamburg, Germany). The fold change was calculated using the 2−ΔΔCt method after normalization to GAPDH. The primers were 5′-CCA AGG TCA TCC ATG ACA AC-3′ and 5′-TCC ACA GTC TTC TGA GTG GC-3′ for GAPDH [29], 5′-GGC TCA GCA CTG CTA TGT TGC C-3′ and 5′-AGC ATG TGG GTC TGG CTG ACT G-3′ for IL-10 [49], 5′-CTT TCC GCG ACA GAG CCT-3′ and 5′-CCA GCT CCA CAC GTC TAT GG-3′ for the β-endorphin precursor proopiomelanocortin (POMC) (NM_139326.2), and 5′-CCT GTC CTT GTG TTC CCT GT-3′ and 5′-AGA GGC AGT CAG GGT GAG AA-3′ for the dynorphin precursor prodynorphin (PDYN) [32]. All the primers were synthesized by Synbio Technologies (Suzhou, China).

### Protein extraction and Western blotting

Cultured primary microglia were seeded in 6-well plates. The cells were treated with GW9508 (100 μM) for about 30 min on the next day, and then harvested with cold PBS and lysed in the radioimmunoprecipitation assay (RIPA) lysis buffer with the addition of the phosphates inhibitor cocktail and the protease inhibitor cocktail (Biotool, Houston, USA) after being centrifuged. Cell lysates were denatured at 100 °C for 10 min and separated in 12% sodium dodecyl sulfatepolyacrylamide gel electrophoresis (SDS-PAGE) gels and then transferred to a PVDF membrane. The membrane was blocked with 5% skim milk in 1 × TBS containing 0.1% Tween-20 (TBST) and incubated with different primary antibodies against phospho-p38, phospho-ERK1/2, phospho-JNK (1:1000, rabbit polyclonal, Cell Signaling Technology, Danvers, MA, USA), and GAPDH (1:2500, mouse monoclonal, Proteintech Group, Rosemont, IL, USA) diluted in the antibody diluent (Beyotime Biotechnology, Shanghai, China) at 4 °C overnight. On the following day, the membrane was thoroughly washed in TBST for 4 times (10 min each time) and further incubated with the Dylight 680-conjugated anti-mouse IgG and Dylight 800-conjugated anti-rabbit IgG (H + L) (Cell Signaling Technology) at room temperature for 1 h. The fluorescent density of each protein band was scanned by using the Odyssey Infrared Imaging System (Li-Cor Biosciences, Lincoln, NE, USA) after being washed 4 times in TBST over 40 min. The relative expression of each protein was analyzed and quantified by using the ImageJ software (National Institutes of Health, Bethesda, MD, USA) for the band ratios of phospho-p38/GAPDH, phospho-ERK1/2/GAPDH, and phospho-JNK/GAPDH.

### Immunofluorescence staining

Double immunofluorescence staining of GPR40, β-endorphin or IL-10, and cellular biomarkers of microglia, astrocytes, and neurons was undertaken and observed using a TCS SP8 confocal microscope (Leica Microsystems, Wetzlar, Germany) in cultured primary cells and the spinal cord sections using modified protocols [29].

For immunocytochemistry, cultured primary microglia, astrocytes, and neurons were seeded on poly-l-lysine pre-coated coverslips placed at the bottom of 12-well plates (5 × 10^4^ cells/well). After culture overnight, cells were washed with PBS and fixed in cool 4% paraformaldehyde for 1 h and were further blocked in the PBS containing 10% goat serum and 0.5% Triton X-100 for 1 h after being washed three times for 15 min in total with PBS. The cell flasks were then incubated with various primary antibodies (Table 1) in blocking buffer at 4 °C overnight, followed by incubation with Alexa 555-conjugated goat anti-rabbit secondary antibody (1:200; Life Technologies, Carlsbad, CA, USA) and Alexa 488-conjugated goat anti-mouse secondary antibody (1:200; Life Technologies) for 1 h at 37 °C. Nucleic dye reagent 2-(4-Amidinophenyl)-6-indolecarbamidine dihydrochloride (DAPI, 0.1 μg/ml; Beyotime, Shanghai, China) was used to stain cell nuclei. The expression of GPR40, Iba-1, GFAP, and NeuN was visualized under a laser scanning confocal microscope. Colocalization analysis was carried out by utilizing a computer-assisted image analysis program (ImageJ Software, National Institutes of Health, USA) equipped with a colocalization finder, under which colocalized pixels appeared as white points [29].

**Table 1:** Dilution ratios of primary antibodies against GPR40, IL-10, β-endorphin, Iba-1, NeuN, and GFAP in immunocytochemistry and immunohistochemistry

Primary antibody	Manufacturer	Dilution ratio	
		Cell flask	Tissue section
GPR40	Santa Cruz Biotech, Santa Cruz, CA, USA	1:100	1:50
IL-10	R&D system, Minneapolis, MN, USA	–	1:200
β-Endorphin	Phoenix Pharmaceuticals, Burlingame, CA, USA	–	1:100
Iba-1	Merck Millipore, Darmstadt, Germany	1:300	1:100
NeuN	Merck Millipore, Darmstadt, Germany	1:100	1:60
GFAP	Merck Millipore, Darmstadt, Germany	1:200	1:100

For immunohistochemistry, rats were anesthetized by intraperitoneal pentobarbital sodium (40 mg/kg) and then intracardially perfused with 100 ml normal saline, followed by 60 ml of 4% paraformaldehyde. The spinal lumbar enlargements (L3–5) were isolated and fixed in paraformaldehyde for 12 h and dehydrated in gradient sucrose solutions (10%, 20%, and 30% in PBS) at 4 °C until precipitated at the bottom. Tissue sections were embedded in the OCT embedding agent (Leica Microsystems) and cut into 30-μm slices. The tissue sections were then incubated with 10% goat serum (*v*/*v*) and 0.5% Triton X-100 (*v*/*v*) in PBS for 1 h. The following steps were the same as the immunocytochemistry, with various primary antibodies (Table 1) and secondary antibodies.

To quantify the relative intensity of GPR40, IL-10, and β-endorphin or their co-expression in microglia, astrocytes, and neurons in the spinal cord, photomicrographs of the medial threefourths of the dorsal horn (laminas I–V) were captured under × 10 or × 30 magnification. The positively stained surface area was measured by using the ImageJ Software after the low and high thresholds were set to exclude the background fluorescence. Only immunofluorescent intensity measurements from positively stained areas were included. The same setup configurations were used to measure all the surface areas in each experimental group at the same time. The averaged value of the positive immunofluorescence area fraction from three non-adjacent sections of each spinal cord was recorded as the relative expression or co-expression.

### IL-10 and β-endorphin measurement by ELISA kits

IL-10 and β-endorphin were measured both in primary cells and spinal enlargements of neuropathic rats. For primary cells, cell culture supernatants were collected after treatment with GW9508 for 2 h and then centrifuged at 5000 rpm at 4 °C for 5 min. The supernatants after being centrifuged were prepared as the protein sample for ELISA. For the spinal enlargements, they were isolated from neuropathic rats after drug administrations, homogenized at 4000 rpm for 15 s with a homogenizer (Fluko Equipment) in 10 mM Tris-HCl (1 g of tissue/5 ml), and centrifuged at 4000 rpm at 4 °C for 15 min. The total protein concentration was measured by using the standard BCA assay (Beyotime Institute of Biotechnology, Shanghai, China). The levels of IL-10 and β-endorphin were measured using commercial IL-10 ELISA kit (Invitrogen) and β-endorphin ELISA kit (Phoenix Pharmaceuticals) according to the operation manuals. The concentrations of IL-10 and β-endorphin were calculated by a calibration curve performed at the same time. The linear range was 1–500 and 1–100 pg/ml for the IL-10 and β-endorphin assay, respectively.

### Experimental design and statistical analyses

For the behavioral study, 5–6 male rats were randomly assigned to each group, and the behavioral measurements were carried out at different time points. For the analysis of dose-response curve, the percentage of maximal possible effect (% MPE) was calculated using the following formula: (post-drug threshold in ipsilateral hindlimb – pre-drug threshold in ipsilateral hindlimb) × 100%/(post-drug threshold in contralateral hindlimb – pre-drug threshold in ipsilateral hindlimb). The % MPE values approximated to 100 indicate normal mechanical thresholds or thermal latency (i.e., near contralateral thresholds), while values approximated to 0 indicate mechanical allodynia or thermal hyperalgesia. Half-effective dose (ED_50_) were calculated by using GraphPad Prism (Version 7.0, GraphPad Software, Inc., San Diego, CA, USA) by fitting nonlinear least squares curves to the relation *Y* = *a* + *bx*, where *x* = [*D*]^*n*^/(ED_50_^*n*^ + [*D*]^*n*^). The values of ED_50_ and *b* (*E*_max_) were projected by yielding a minimum residual sum of squares of deviations from the theoretical curve [50].

Results were summarized as means ± SEM or 95% confidence intervals. The analysis of variance was performed by unpaired and two-tailed Student’s *t* test and one-way or repeated measures two-way ANOVA using GraphPad Prism to determine the significant difference. The post hoc Student-Newman-Keuls test was conducted when the effect of the drug (dose) (for one-way ANOVA, the factor was drug [dose]; for two-way ANOVA, the factors were drug [dose], time, and their interaction) was statistically significant. Values of *P* < 0.05 were considered statistical significance.

## Results

### Intrathecal GW9508 exerted mechanical antiallodynia and thermal antihyperalgesia in neuropathic pain via GPR40 activation

**Fig. 1:**
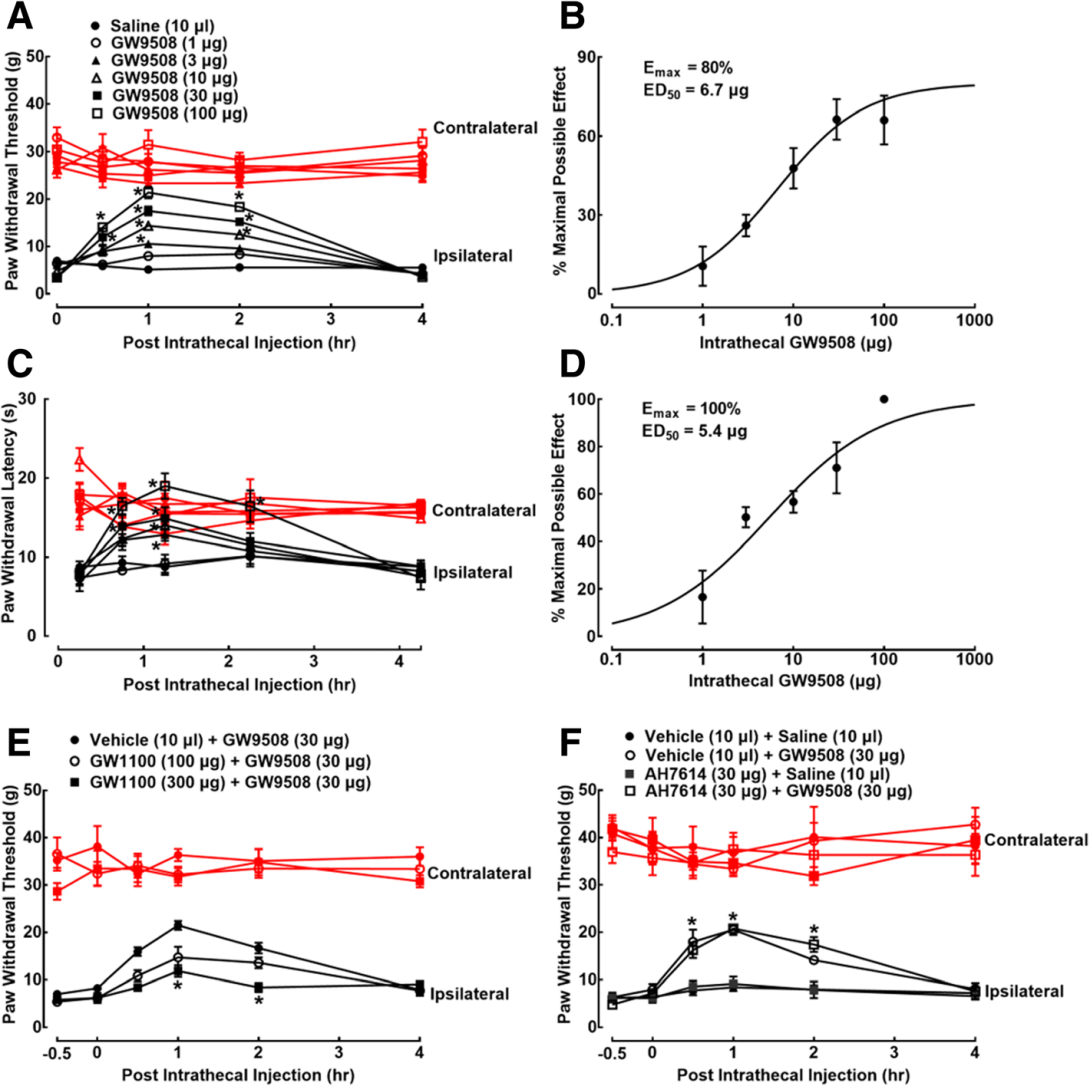
Inhibitory effects of GW9508 given intrathecally on mechanical allodynia (**a**, **b**) and thermal hyperalgesia (**c**, **d**) in neuropathic rats induced by spinal nerve ligation. Dose-response analyses of GW9508 on mechanical allodynia (**b**) and thermal hyperalgesia (**d**) were calculated using the thresholds measured 1 h after injection, best projected by the nonlinear least squares method. Effects of the selective GPR40 antagonist GW1100 (**e**) and GPR120 antagonist AH7614 (**f**) on GW9508-induced mechanical antiallodynia. Data are presented as means ± SEM (*N* = 6~7 per group). *P < 0.05 vs. the vehicle control group; analyzed by repeated measures two-way ANOVA followed by the post hoc Student-Newman-Keuls test

The antinociceptive effects of the GPR40 agonist GW9508 were assessed in neuropathic rats induced by L5/L6 spinal nerve ligation approximately 2 weeks after surgery. Six groups of neuropathic rats received an intrathecal injection of either normal saline (10 μl) or GW9508 (1, 3, 10, 30, or 100 μg). The hindpaw withdrawal thresholds to the von Frey monofilament stimulus and paw withdrawal latencies to the thermal stimulus were subsequently (in a 10-min interval) measured before and 0.5, 1, 2 and 4 h post-intrathecal injection. In normal saline-treated rats, withdrawal thresholds in either contralateral or ipsilateral hindpaws remained unchanged during the observation period of 4 h. In contrast, the intrathecal injection of GW9508 attenuated mechanical allodynia in ipsilateral hindpaws in a dose- and time-dependent manner, without significantly affecting withdrawal thresholds in contralateral hindpaws (Fig. 1a). Mechanical thresholds at 1 h after injection were used for % MPE calculation and subsequent dose-response analysis. The projected *E*_max_ and ED_50_ values for mechanical antiallodynia inhibition were 80% MPE and 6.7 μg (95% confidence intervals 4.1–10.8 μg) (Fig. 1b). Furthermore, the intrathecal injection of GW9508 also dose- and time-dependently reduced the thermal hyperalgesia in ipsilateral hindpaws (Fig. 1c), with the projected *E*_max_ value of 100% MPE and ED_50_ value of 5.4 μg (95% confidence intervals 3.14–9.30 μg) (Fig. 1d).

GW9508 is also an agonist of GPR120, although with the affinity is 100-fold less than that of GPR40 [51]. The selective GPR40 antagonist GW1100 and GPR120 antagonist AH7614 were used to confirm whether or not GW9508 attenuated neuropathic pain through the activation of GPR40. Three groups of neuropathic rats received intrathecal injection of the vehicle (30% DMSO, 70% PEG400, 10 μl) and GW1100 (100 and 300 μg), respectively, followed by the second injection of GW9508 (30 μg) 30 min later. Paw withdrawal thresholds were measured before and 0.5, 1, 2, and 4 h post-GW9508 administration. Intrathecal injection of GW9508 exerted the time-dependent mechanical antiallodynia. Although GW1100 did not significantly alter baseline withdrawal responses in either hindpaws, it dose-dependently reversed GW9508-induced mechanical antiallodynia (*P* < 0.05, by repeated measures two-way ANOVA followed by the post hoc Student-Newman-Keuls test; Fig. 1e). In addition, four groups of neuropathic rats were intrathecally administrated with the vehicle (10 μl) + saline (10 μl), vehicle (10 μl) + GW9508 (30 μg), AH7614 (30 μg) + saline (10 μl), and AH7614 (30 μg) + GW9508 (30 μg). The first intrathecal injection was followed by the second injection 30 min later. Intrathecal injection of GW9508 produced mechanical antiallodynia time-dependently (*P* < 0.05, by repeated measures two-way ANOVA followed by the post hoc Student-Newman-Keuls test), which was not significantly altered by pretreatment with intrathecal AH7614 (Fig. 1f).

### Intrathecal GW9508 stimulated IL-10 and β-endorphin expression in the spinal dorsal horn

**Fig. 2:**
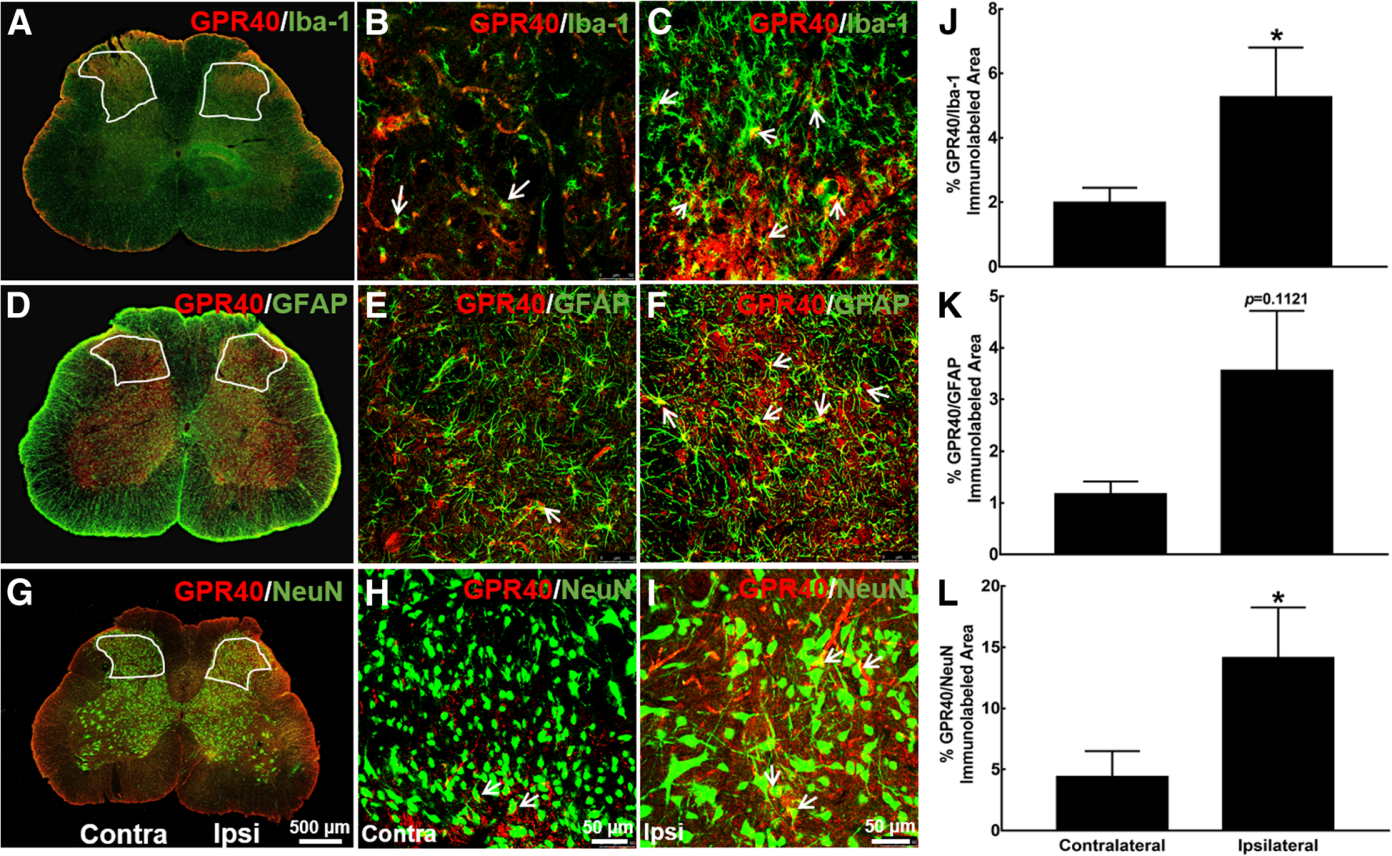
Expression of GPR40 in microglia (**a**–**c**), astrocytes (**d**–**f**), and neurons (**g**–**i**) in the spinal dorsal horn of neuropathic rats. Frozen sections of the spinal lumbar enlargements were obtained approximately 2 weeks after spinal nerve ligation. Immunofluorescence was double stained with GPR40/Iba-1, GPR40/GFAP, and GPR40/NeuN, and photomicrographs were taken from the spinal cord section (**a**, **d**, **g**; 500 μm) and amplified dorsal horn laminae I–V (**b**, **c**, **e**, **f**, **h**, **i**; 50 μm). Arrows indicate double immunostaining of GPR40 with each cellular biomarker. Double immunolabeled surface areas of GPR40/Iba-1 (**j**), GPR40/GFAP (**k**), and GPR40/NeuN (**l**) from the spinal dorsal horn laminae I–V indicated in white lines were quantified using the ImageJ program. Data are presented as means ± SEM (*N* = 5–6 per group). *P < 0.05 vs. contralateral side; analyzed by unpaired and two-tailed Student’s *t* test

GPR40 shows diversified distributions in the brain, pancreatic islets, spinal cord, pituitary, liver, heart, and skeletal muscle [2–4, 52]. We first examined its expression in the spinal cords of neuropathic rats by utilizing double immunofluorescence staining. Frozen sections were obtained from the spinal lumbar enlargements of neuropathic rats induced by spinal nerve ligation approximately 2 weeks after surgery. Immunofluorescence was stained with antibodies against GPR40 with cellular biomarkers for microglia, astrocytes, and neurons (Iba-1, GFAP, or NeuN, respectively). Photomicrographs were taken from the entire spinal cord (× 10 magnification) and dorsal horn laminae I–V (× 30 magnification). As shown in Additional file 1: Figure S1A, GPR40-positive cells were mainly expressed in the contralateral and ipsilateral dorsal horns, as well as in the ventral horns. In addition, double immunofluorescence staining of GPR40 with Iba-1, GFAP, and NeuN revealed that GPR40 was colocalized with microglia, astrocytes, and neurons in the contralateral and ipsilateral dorsal horn I–V laminae. Interestingly, the ipsilateral dorsal horn exhibited much higher expression of GPR40 immunostaining on all of microglia, astrocytes, and neurons, compared to the contralateral side (Fig. 2a–i). The ImageJ program was used to quantify immunofluorescence intensity (immunolabeled surface area) in the dorsal horn (× 10 magnification). As shown in Additional file 1: Figure S1B, the GPR40-immunolabeled surface areas in the ipsilateral spinal dorsal horn were significantly increased by 102%, compared with the contralateral side (*P* < 0.05, by unpaired and two-tailed Student’s *t* test). Double immunostaining of GPR40/Iba-1, GPR40/GFAP, and GPR40/NeuN in the ipsilateral dorsal horn was significantly increased by 165%, 203%, and 219%, respectively (*P* < 0.05, by unpaired and two-tailed Student’s *t* test; Fig. 2j–l).

**Fig. 3:**
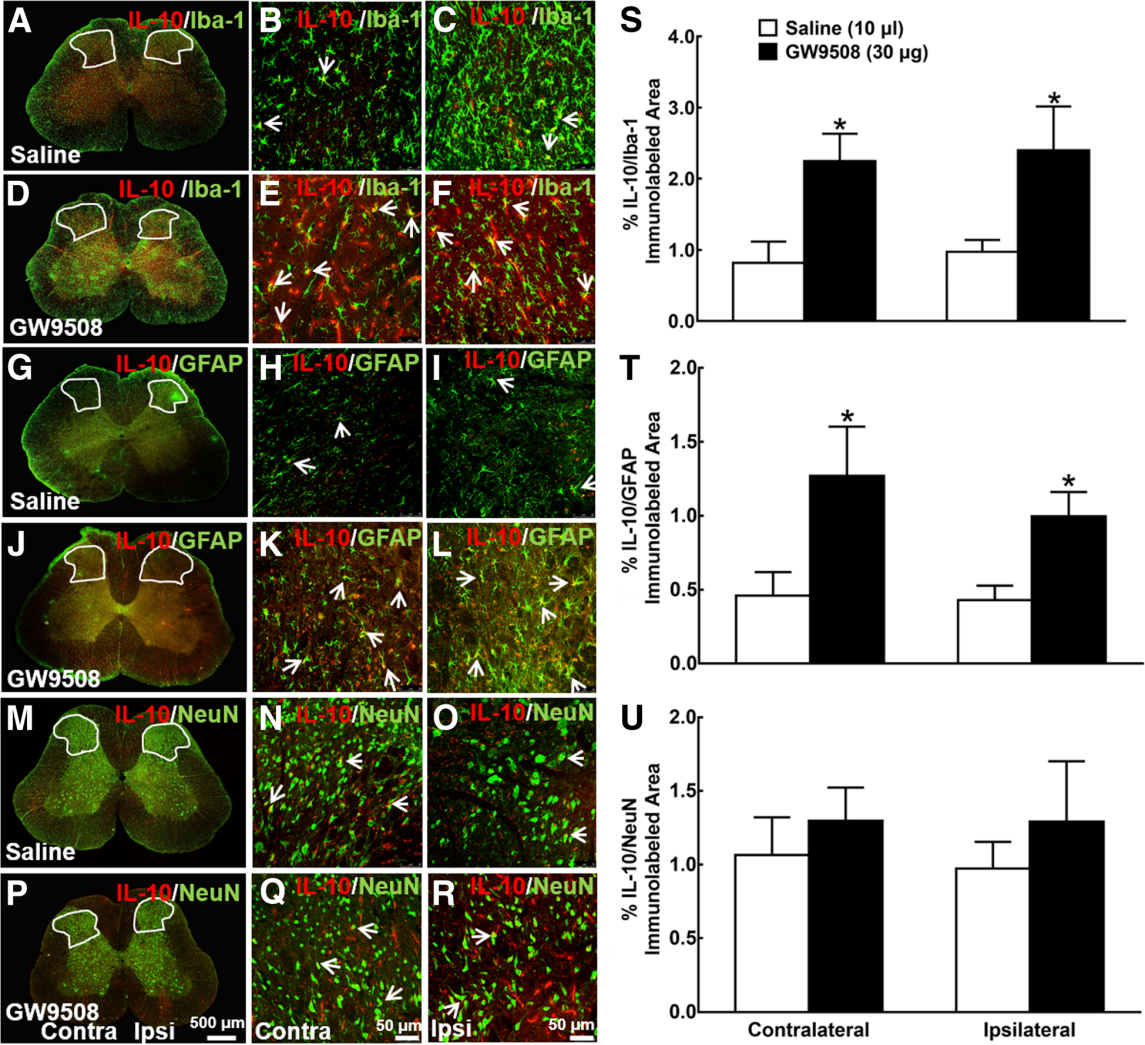
Effect of intrathecal GW9508 on spinal IL-10 expression on spinal microglia (**a**–**f**), astrocytes (**g**–**l**), and neurons (**m**–**r**) in neuropathic rats induced by spinal nerve ligation. Frozen sections of the spinal lumbar enlargements were obtained 1 h after intrathecal saline (10 μl) or GW9508 (30 μg) treatment. Immunofluorescence was double stained with IL-10/Iba-1, IL-10/GFAP, and IL-10/NeuN, and photomicrographs were taken from the entire spinal cord section (**a**, **d**, **g**, **j**, **m**, **p**; 500 μm) and amplified dorsal horn laminae I–V (**b**, **c**, **e**, **f**, **h**, **i**, **k**, **l**, **n**, **o**, **q**, **r**; 50 μm). Arrows indicate double immunostaining of IL-10 and each cellular biomarker. Double immunolabeled surface areas of IL-10/Iba-1 (**s**), IL-10/GFAP (**t**), and IL-10/NeuN (**u**) from the spinal dorsal horn laminae I-V indicated in white lines were quantified using the ImageJ program. Data are presented as means ± SEM (*N* = 5~7 per group). *P < 0.05 vs. saline group; analyzed by unpaired and two-tailed Student’s *t* test

We then assessed the stimulatory effects of GW9508 given intrathecally on the spinal level of IL-10 and its cell localization. Spinal enlargements were isolated from neuropathic rats 1 h after intrathecal normal saline (10 μl) or GW9508 (30 μg) treatment for IL-10 measurement. As shown in Additional file 2: Figure S2A and S2B, both gene and protein levels of IL-10 were significantly increased by the intrathecal injection of GW9508 (*P* < 0.05, by unpaired and two-tailed Student’s *t* test). Moreover, frozen sections were obtained 1 h after intrathecal normal saline (10 μl) or GW9508 (30 μg) treatment. Immunofluorescence was stained with antibodies against GPR40 with Iba-1, GFAP, and NeuN. In neuropathic rats treated with intrathecal normal saline, IL-10 was widely spread in the gray matter of both contralateral and ipsilateral spinal dorsal horns and in the ventral horns, which was markedly enhanced by the intrathecal administration of GW9508 (30 μg) in both contralateral and ipsilateral sides (Additional file 2: Figure S2C and S2D). Double immunofluorescence staining further revealed that IL-10 was colocalized with Iba-1 in the dorsal horn of saline-treated rats (Fig. 3a–c). Intrathecal injection of GW9508 significantly enhanced the co-immunostaining of IL-10/Iba-1 in both contralateral and ipsilateral dorsal horns (Fig. 3d–f). In addition, IL-10 was also colocalized with GFAP (Fig. 3g–i) and the intrathecal injection of GW9508 significantly enhanced the double immunostaining of IL-10/GFAP in both contralateral and ipsilateral dorsal horns (Fig. 3j–l). In contrast, although IL-10 was colocalized with NeuN (Fig. 3m–o), the intrathecal GW9508 failed to significantly increase the immunostaining of IL-10/NeuN either in the contralateral or ipsilateral dorsal horn (Fig. 3p–r).

The ImageJ program was used to quantify immunofluorescence intensity in the dorsal horn. As shown in Additional file 2: Figure S2E, the intrathecal GW9508 significantly increased the IL-10-immunolabeled surface areas by 520% and 380% in the contralateral and ipsilateral dorsal horns, compared with saline group (*P* < 0.05, by unpaired and two-tailed Student’s *t* test). Furthermore, GW9508 significantly increased the double immunostaining of IL-10/Iba-1 by 290% and 200%, respectively, in the contralateral and ipsilateral dorsal horns (*P* < 0.05, by unpaired and two-tailed Student’s *t* test; Fig. 3s). Although the baseline double immunostaining intensity of IL-10/GFAP was much lower than that of IL-10/Iba-1, it was significantly increased by GW9508 injection by 280% and 230% in contralateral and ipsilateral spinal dorsal horns, respectively (*P* < 0.05, by unpaired and two-tailed Student’s *t* test; Fig. 3t). However, GW9508 failed to significantly increase the double immunostaining intensity of IL-10/NeuN in either contralateral or ipsilateral spinal dorsal horn (Fig. 3u).

**Fig. 4:**
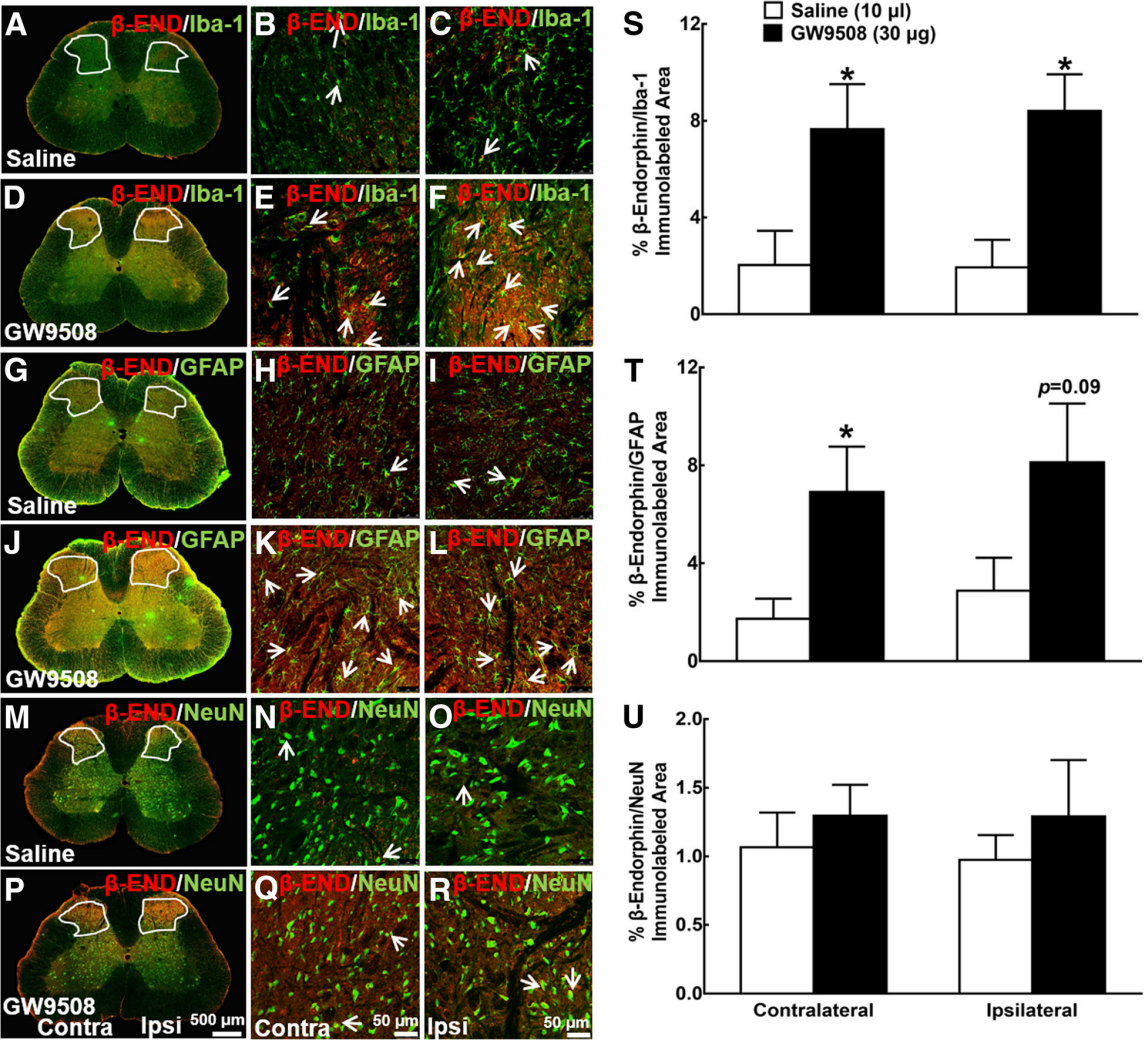
Effect of GW9508 given intrathecally on spinal β-endorphin expression on spinal microglia (**a**–**f**), astrocytes (**g**–**l**), and neurons (**m**–**r**) in neuropathic rats induced by spinal nerve ligation. Frozen sections of the spinal lumbar enlargements were obtained 1 h after intrathecal saline (10 μl) or GW9508 (30 μg) treatment. Immunofluorescence was double stained with β-endorphin/Iba-1, β-endorphin/GFAP, and β-endorphin/NeuN, and photomicrographs were taken from the entire spinal cord section (**a**, **d**, **g**, **j**, **m**, **p**: 500 μm) and amplified dorsal horn laminae I–V (**b**, **c**, **e**, **f**, **h**, **i**, **k**, **l**, **n**, **o**, **q**, **r**: 50 μm). Arrows indicate double immunostaining of β-endorphin with each cellular biomarker. Double immunolabeled surface areas of β-endorphin/Iba-1 (**s**), β-endorphin/GFAP (**t**), and β-endorphin/NeuN (**u**) from the spinal dorsal horn laminae I-V indicated in white lines were quantified using the ImageJ program. Data are presented as means ± SEM (*N* = 5~7 per group). *P < 0.05 vs. saline group; analyzed by unpaired and two-tailed Student’s *t* test

We further assessed the stimulatory effect of intrathecal GW9508 treatment on β-endorphin expression in the above same spinal enlargement samples and spinal slides. β-Endorphin was markedly enhanced by the intrathecal GW9508 in both gene and protein level as well (Additional file 3: Figure S3A and S3B). β-Endorphin was also widely spread in the gray matter of both contralateral and ipsilateral spinal dorsal horns and ventral horns from the control neuropathic rats, and the intrathecal GW9508 injection significantly enhanced the spinal β-endorphin expression (Additional file 3: Figure S3C and S3D). β-Endorphin was colocalized with Iba-1 in the dorsal horns of control rats (Fig. 4a–c). Intrathecal injection of GW9508 significantly increased the double immunostaining of β-endorphin/Iba-1 both in the contralateral and ipsilateral spinal dorsal horns (Fig. 4d–f). In addition, β-endorphin was also colocalized with GFAP (Fig. 4g–i) and the intrathecal injection of GW9508 significantly enhanced its co-immunostaining in both contralateral and ipsilateral dorsal horns (Fig. 4j–l). In contrast, although β-endorphin was also colocalized with NeuN (Fig. 4m–o), the intrathecal GW9508 failed to significantly increase its double immunostaining in either the contralateral or ipsilateral dorsal horn (Fig. 4p–r). The ImageJ quantification in the dorsal horn showed that the intrathecal GW9508 significantly increased the immunofluorescence intensity of β-endorphin by 700% and 800% (*P* < 0.05, by unpaired and two-tailed Student’s *t* test; Additional file 3: Figure S3E), double immunofluorescence intensity of β-endorphin/Iba-1 by 380% and 430% (*P* < 0.05, by unpaired and two-tailed Student’s *t* test; Fig. 4s), and β-endorphin/GFAP by 360% and 260% (*P* = 0.06 and 0.13, by unpaired and two-tailed Student’s *t* test; Fig. 4t) in contralateral and ipsilateral spinal dorsal horn, respectively, compared with the saline group. In contrast, the intrathecal GW9508 failed to stimulate the double immunostaining of β-endorphin/NeuN in either contralateral or ipsilateral spinal dorsal horn (Fig. 4u).

### GW9508 stimulated IL-10 and β-endorphin expression in cultured primary microglia and astrocytes

**Fig. 5:**
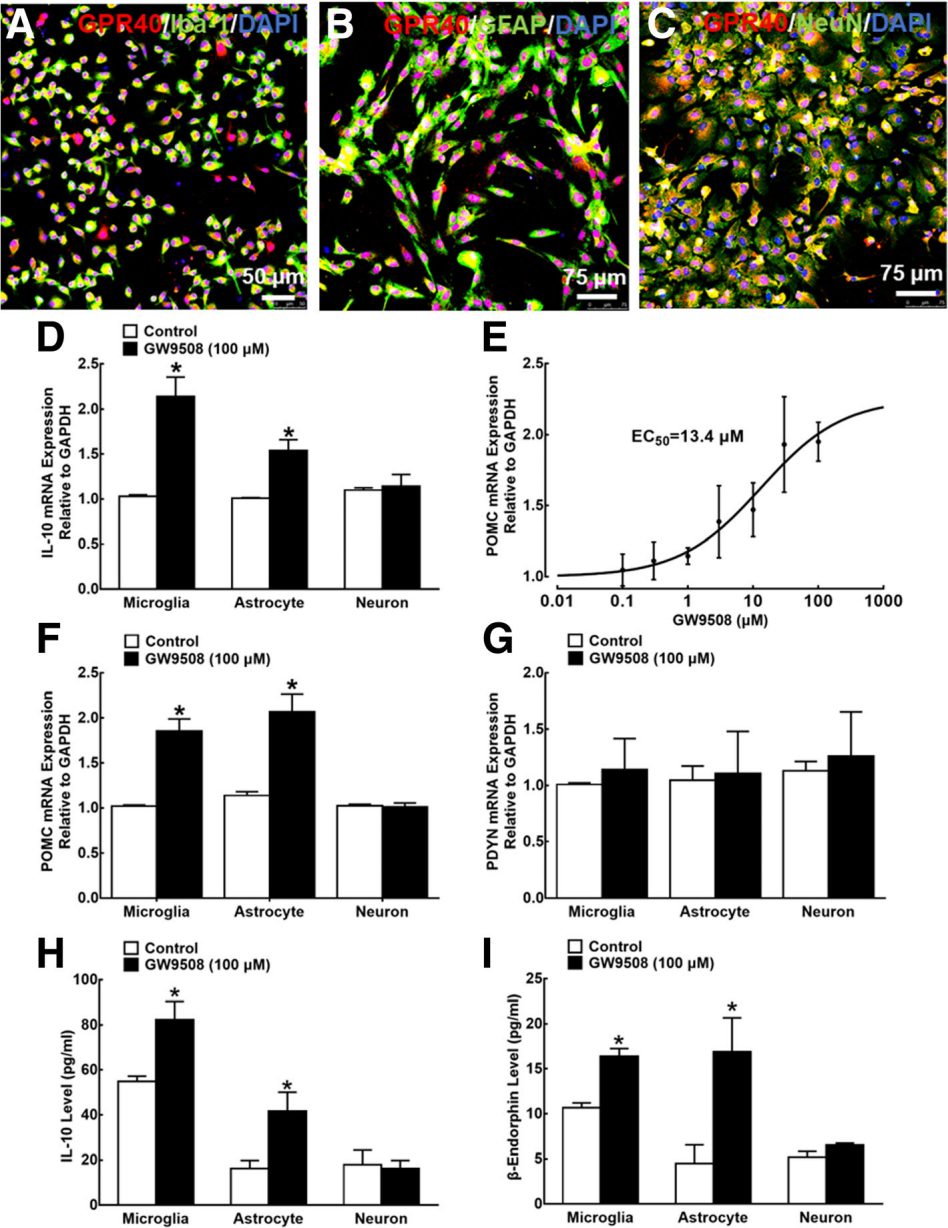
Expression of GPR40 (**a**–**c**) and stimulatory effects of GW9508 on mRNA expression of IL-10, the β-endorphin precursor proopiomelanocortin (POMC) and dynorphin A precursor prodynorphin (PDYN) (**d**–**g**), and secretion of IL-10 and β-endorphin (**h**, **i**) in cultured primary microglia, astrocytes, and neurons originated from the spinal cords of 1-day-old neonatal rats. For the immunostaining study, immunofluorescence was double stained with GPR40/Iba-1, GPR40/GFAP, and GPR40/NeuN. For the stimulatory study, cultured cells and cultural medium were collected 2 h after GW9508 incubation, and the mRNA expression of IL-10, POMC, and PDYN and the peptide concentrations of IL-10 and β-endorphin were measured using quantitative real-time PCR and commercial ELISA kits, respectively. Data are presented as means ± SEM (*N* = 4~7 per group). *P < 0.05 vs. control group; analyzed by unpaired and two-tailed Student’s *t* test

To further assess the cell expression of GPR40, microglia, astrocytes, and neurons were isolated from the spinal cords of neonatal rats and were co-immunostained with GPR40/Iba-1, GPR40/GFAP, and GPR40/NeuN, respectively, in addition to the nucleus staining with DAPI. As shown in Fig. 5a–c, GPR40 was expressed on microglia, astrocytes, and neurons, with 100% positive in each cell population.

We then tested the stimulatory effect of GPR40 activation on IL-10 and β-endorphin expression. Primary microglia, astrocytes, and neurons from the spinal cords of neonatal rats were incubated with 100 μM GW9508 for 2 h, and the cellular mRNA expression of IL-10, POMC, and PDYN was measured by using quantitative real-time PCR. Incubation with GW9508 for 2 h significantly increased the expression of IL-10 mRNA in cultured primary microglia and astrocytes (*P* < 0.05, by unpaired and two-tailed Student’s *t* test), but not neurons (Fig. 5d). Further analysis showed that the GW9508 treatment concentration dependently increased the POMC expression in microglia, with an EC_50_ value of 13.4 μM (Fig. 5e). In addition, the treatment with GW9508 for 2 h significantly increased the mRNA expression of POMC in cultured primary microglia and astrocytes (*P* < 0.05, by unpaired and two-tailed Student’s *t* test), but not in neurons (Fig. 5f). In contrast, GW9508 failed to affect the PDYN mRNA expression in microglia, astrocytes, or neurons (Fig. 5g).

The secretory effects of GW9508 on IL-10 and β-endorphin were also investigated. Primary cultures of microglia, astrocytes, and neurons were incubated with 100 μM GW9508 for 2 h, and the concentration of IL-10 and β-endorphin in the cultural medium were measured using commercial ELISA kits. As exhibited in Fig. 5h and i, the incubation with GW9508 for 2 h significantly increased the IL-10 and β-endorphin secretion from microglia and astrocytes (*P* < 0.05, by unpaired and two-tailed Student’s *t* test). However, the treatment with GW9508 did not affect the IL-10 or β-endorphin secretion from neurons.

**Fig. 6:**
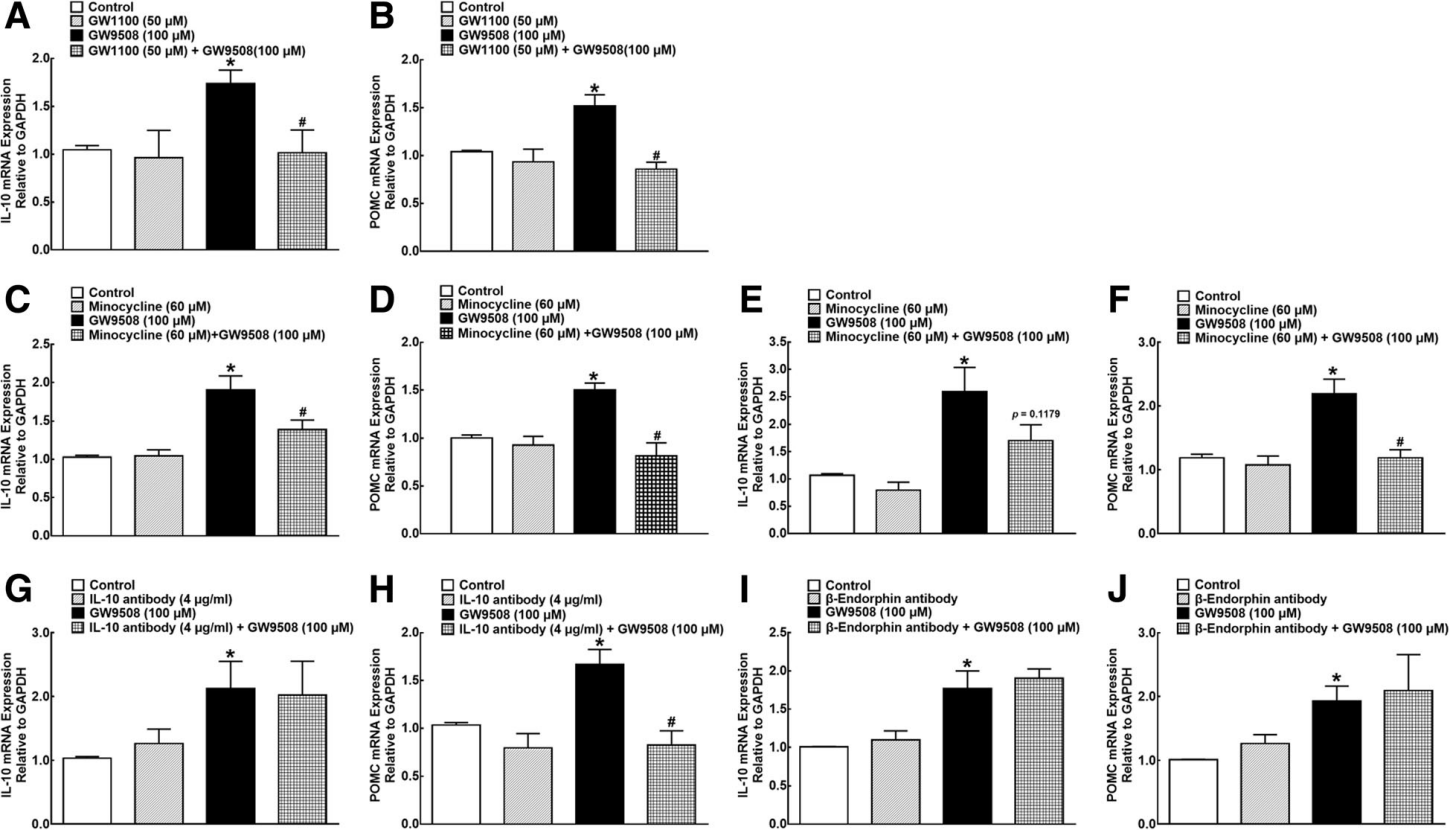
Effects of the specific GPR40 antagonist GW1100 (**a**, **b**), glial inhibitor minocycline (**c**–**f**), IL-10 (**g**, **h**), and β-endorphin (**i**, **j**) neutralizing antibodies on the GW9508-enhanced mRNA expression of IL-10 and the β-endorphin precursor proopiomelanocortin (POMC) in microglia and astrocytes. Cultured primary microglial cells (**a**–**d**, **g**–**j**) and astrocytes (**e**, **f**), originated from the spinal cords of neonatal rats, were collected 2 h after drug treatment, and the mRNA expression of IL-10 and POMC was determined using quantitative real-time PCR. Data are presented as means ± SEM (*N* = 4~8 per group). Asterisk (*) and number sign (#) indicate *P* < 0.05 vs. the vehicle control group and GW9508 group, respectively; analyzed by one-way ANOVA followed by the post hoc Student-Newman-Keuls test

To confirm the stimulatory effect of GW9508 on IL-10 and β-endorphin expression through GPR40, the selective GPR40 antagonist GW1100 was utilized. As shown in Fig. 6a and b, the pretreatment with GW1100 (50 μM) for 30 min did not significantly the change basal expression of IL-10 or POMC mRNA in the cultured primary microglia, but totally blocked the GW9508 (100 μM)-induced elevation of IL-10 and POMC mRNA (*P* < 0.05, by one-way ANOVA followed by the post hoc Student-Newman-Keuls test).

Minocycline is generally considered as a microglia inhibitor [53–55], but may inhibit astrocyte activation as well [56–58]. To confirm its glial inhibitory effect, minocycline was used to block the GW9508-induced mRNA expression of IL-10 and POMC in microglia and astrocytes. Pretreatment with minocycline (60 μM) in the cultured primary microglia for 1 h did not change the baseline expression of IL-10 and POMC mRNA, but entirely blocked the GW9508 (100 μM)-elevated IL-10 and POMC mRNA expression (*P* < 0.05, by one-way ANOVA followed by the post hoc Student-Newman-Keuls test; Fig. 6c, d). Moreover, as exhibited in Fig. 6e and f, GW9508 also markedly increased the IL-10 and POMC mRNA expression in the cultured primary astrocytes, and the pretreatment with minocycline significantly inhibited the GW9508-stimulated POMC mRNA expression (*P* < 0.05, by one-way ANOVA followed by the post hoc Student-Newman-Keuls test). Minocycline also appeared to inhibit the GW9508-induced IL-10 expression, although it did not reach statistical significance (*P* = 0.12).

We have recently discovered that exogenous IL-10 and endogenous IL-10 after GLP-1 receptor activation induced the β-endorphin expression in microglia [49]. Following GPR40 activation, the IL-10 and β-endorphin neutralizing antibodies were used to illustrate IL-10 to induce β-endorphin and vice versa. As shown above, the treatment with GW9085 (100 μM) significantly increased the IL-10 and POMC mRNA expression in the cultured primary microglia, whereas the treatment with either the IL-10 antibody (4 μg/ml) or β-endorphin antibody (1:300 dilution) had no effects on the baseline expression of both IL-10 and POMC mRNA. The IL-10 neutralizing antibody completely inhibited the GW9508-stimulated mRNA expression of POMC (*P* < 0.05, by one-way ANOVA followed by the post hoc Student-Newman-Keuls test) but not IL-10 (Fig. 6g, h). In contrast, the β-endorphin neutralizing antibody did not exhibit any inhibitory effects on the GW9508-stimulated mRNA expression of either IL-10 or POMC (Fig. 6i, j).

**Fig. 7:**
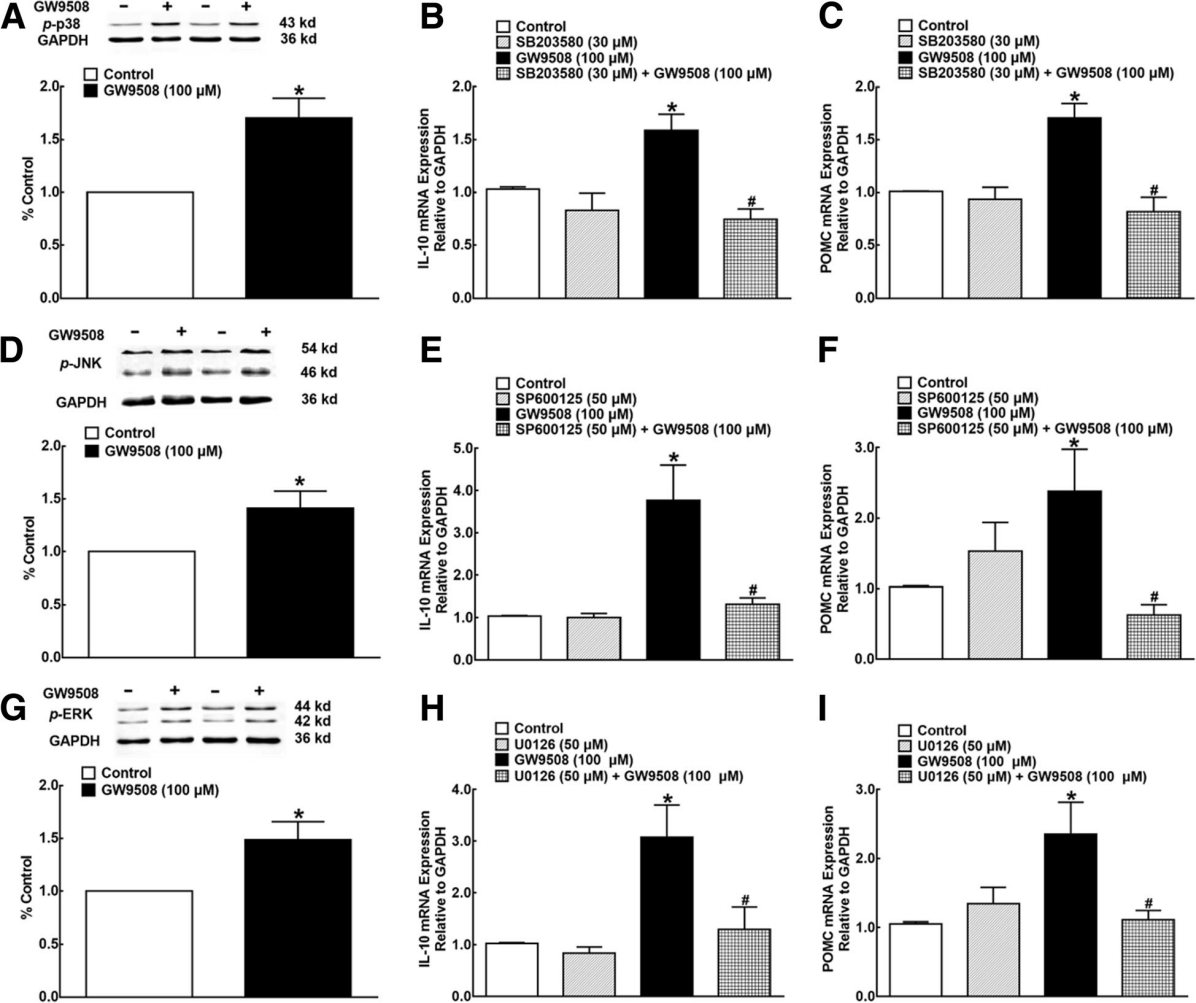
Effects of the phosphorylation of p38 (**a**–**c**), JNK (**d**–**f**), and ERK1/2 mitogen-activated protein kinase (MAPK) (**g**–**i**) on GW9508-enhanced mRNA expression of IL-10 and β-endorphin in microglia. Cultured primary microglial cells were originated from the spinal cords of neonatal rats. For the phosphorylation immunoblotting study, microglia were collected 30 min after the GW9508 treatment, and their lysates were immunoblotted with the antibodies of anti-phospho-p38, anti-phospho-ERK1/2, and anti-phospho-JNK, respectively. Immunoblots of each experiment are placed on the upper panels, and densitometric analyses are shown in the lower panels. For the MAPK inhibitor study, microglia were collected 2 h after drug treatment, and the mRNA expression of IL-10 and the β-endorphin precursor proopiomelanocortin (POMC) was determined using quantitative real-time PCR. Data are presented as means ± SEM (*N* = 5~8 per group). Asterisk (*) and number sign (#) indicate *P* < 0.05 vs. the vehicle control group and GW9508 group, respectively; analyzed by unpaired and two-tailed Student’s *t* test and one-way ANOVA followed by the post hoc Student-Newman-Keuls test

Mitogen-activated protein kinases (MAPKs), including p38, ERK, and JNK, are evolutionarily conserved enzymes that play crucial roles in various cellular processes [59, 60]. We assessed whether GW9508 stimulated IL-10 and POMC mRNA expression through MAPK activation. In cultured primary microglia, the treatment with GW9508 (100 μM) for 30 min significantly increased the p38 phosphorylation by 1.7-fold (*P* < 0.05, by unpaired and two-tailed Student’s *t* test; Fig. 7a). In addition, pretreatment with the selective p38 inhibitor SB203580 (30 μM) significantly inhibited the GW9508-stimulated IL-10 and POMC mRNA expression (*P* < 0.05, by one-way ANOVA followed by followed by the post hoc Student-Newman-Keuls test), although it did not significantly affect their baseline expression (Fig. 7b, c). As shown in Fig. 7d–i, the treatment with GW9508 also stimulated both JNK and ERK1/2 phosphorylation by 1.4- and 1.5-fold, respectively (*P* < 0.05, by unpaired and two-tailed Student’s *t* test), and pretreatment with the selective JNK inhibitor SP600125 (30 μM) and ERK1/2 inhibitor U0126 (30 μM) significantly attenuated the GW9508-increased IL-10 and POMC mRNA expression (*P* < 0.05, by one-way ANOVA followed by the post hoc Student-Newman-Keuls test), without affecting their baseline expression.

### GW9508 exerted antinociception via spinal glial IL-10/β-endorphin pathway

**Fig. 8:**
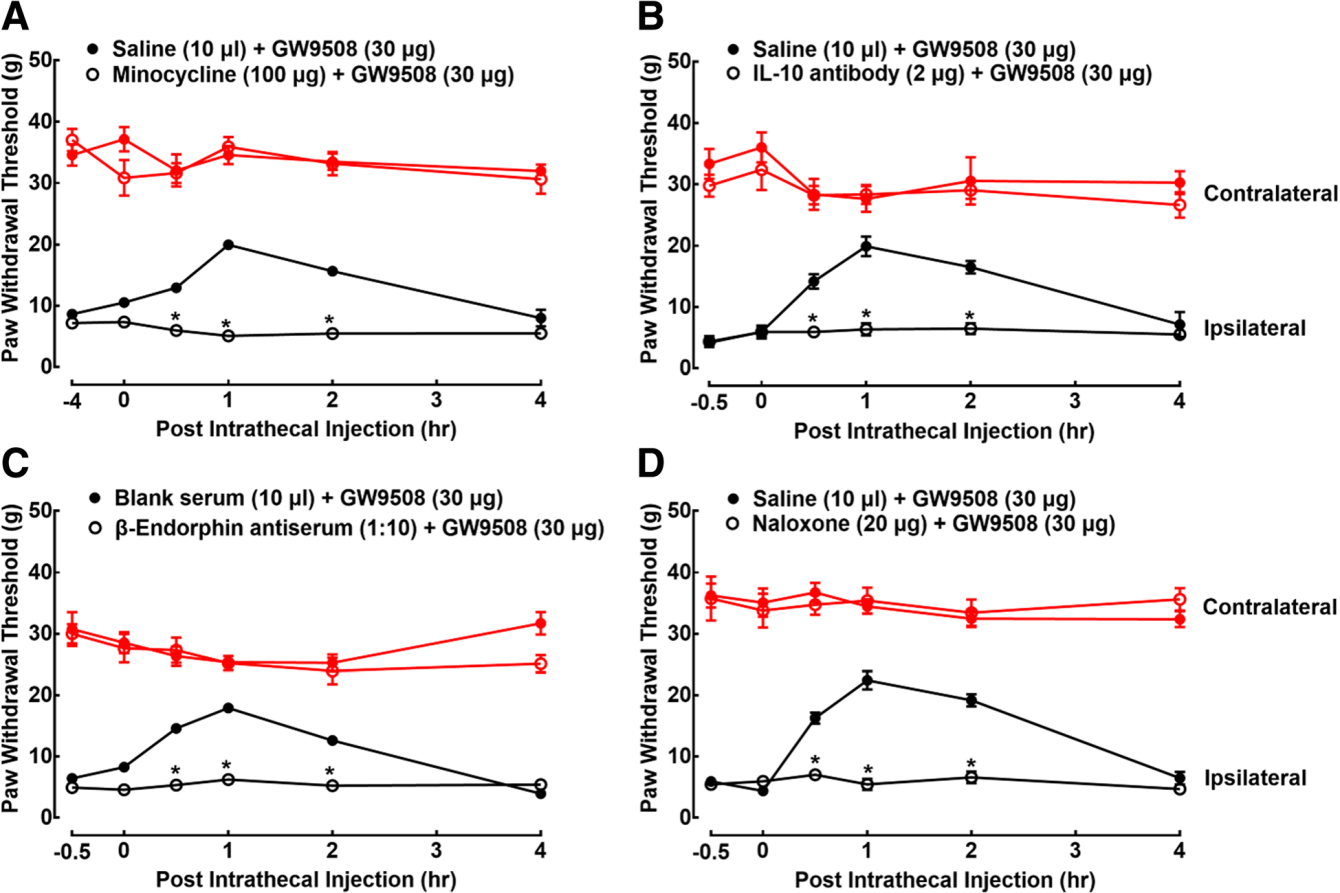
Blockade effects of the glial inhibitor minocycline (**a**), specific IL-10 antibody (**b**), β-endorphin antiserum (**c**), and μ-opioid receptor-preferred antagonist naloxone (**d**), given intrathecally, on the spinal GW9508-induced mechanical antiallodynia in neuropathic rats. Neuropathic rats, 2 weeks after spinal nerve ligation, received a single injection of the vehicle (10 μl) or each blocking agent followed by a single intrathecal injection of GW9508 (30 μg) 4 h (for minocycline) or 30 min (for the rest drugs) later for subsequent assessment of withdrawal responses to mechanical stimuli. Data are presented as means ± SEM (*N* = 5~8 per group). *P < 0.05, vs. vehicle + GW9508 group; analyzed by repeated measures two-way ANOVA followed by the post hoc Student-Newman-Keuls test

Minocycline was used to block the GW9508-induced mechanical antiallodynia. Two groups of neuropathic rats were pretreated with intrathecal saline (10 μl) or minocycline (100 μg) for 4 h followed by the intrathecal injection of GW9508 (30 μg). Paw withdrawal thresholds were measured before and 0, 0.5, 1, 2, and 4 h post-GW9508 administration. The intrathecal injection of GW9508 exerted profound mechanical antiallodynia in a time-dependent manner, which was reversed by the pretreatment with minocycline (*P* < 0.05, by repeated measures two-way ANOVA followed by the post hoc Student-Newman-Keuls test), although it did not alter the baseline withdrawal response in either paw (Fig. 8a).

In order to confirm the causal role of spinal IL-10/β-endorphin in the GW9508-induced mechanical antiallodynia, the IL-10 neutralizing antibody was first applied. Two groups of neuropathic rats received an intrathecal injection of normal saline (10 μl) or the IL-10 antibody (2 μg) for 30 min followed by an intrathecal injection of GW9508 (30 μg). Paw withdrawal thresholds were measured before and 0, 0.5, 1, 2, and 4 h post-GW9508 administration. Pretreatment with intrathecal IL-10 neutralizing antibody completely reversed the mechanical antiallodynia produced by intrathecal GW9508 (*P* < 0.05, by repeated measures two-way ANOVA followed by the post hoc Student-Newman-Keuls test), although it did not significantly alter the baseline withdrawal responses in either contralateral or ipsilateral hindpaws (Fig. 8b).

In addition, two groups of neuropathic rats received an intrathecal injection of the blank serum (1:10 dilution, 10 μl) or the β-endorphin antiserum (1:10 dilution, 10 μl) for 30 min followed by an intrathecal injection of GW9508 (30 μg). Paw withdrawal thresholds were measured before and 0, 0.5, 1, 2, and 4 h post-GW9508 administration. The intrathecal injection of GW9508 exerted profound mechanical antiallodynia in a time-dependent manner, which was completely reversed by the pretreatment with intrathecal β-endorphin antiserum (*P* < 0.05, by repeated measures two-way ANOVA followed by the post hoc Student-Newman-Keuls test), although it did not significantly alter baseline withdrawal responses in either hindpaws (Fig. 8c).

Furthermore, as β-endorphin is an endogenous ligand of μ-opioid receptors [61], the μ-opioid receptor-preferred antagonist naloxone was utilized to illustrate the pivotal role of β-endorphin in the GW9508-induced mechanical antiallodynia. Two groups of neuropathic rats received an intrathecal injection of normal saline (10 μl) or naloxone (20 μg) for 30 min, followed by an intrathecal injection of GW9508 (30 μg). Pretreatment with intrathecal naloxone did not significantly affect baseline withdrawal responses in either hindpaws, but completely blocked the GW9508-exerted mechanical antiallodynia in the ipsilateral paws (*P* < 0.05, by repeated measures two-way ANOVA followed by the post hoc Student-Newman-Keuls test; Fig. 8d).

## Discussion

Our present study demonstrated that the GPR40 agonist GW9508, given intrathecally, was effective in attenuating mechanical allodynia and thermal hyperalgesia in ipsilateral hindpaws of neuropathic rats in a dose-dependent manner, with *E*_max_ values of 80% and 100% MPE and ED_50_ values of 6.7 and 5.4 μg, respectively. It did not significantly affect the normal reflex nociceptive pain states in contralateral hindpaws. We also confirmed that the GW9508-induced mechanical antiallodynia was through GPR40 activation, as the selective GPR40 antagonist GW1100 (but not selective GPR120 antagonist AH7614) dose-dependently and nearly completely suppressed the GW9508-induced antinociception and as the GPR120 was not expressed in the central nervous system including the spinal cord [18]. Our results were in accordance with the previous findings, which showed that the GPR40 agonists DHA, GW9508, and MEDICA16, given orally, intracerebroventricularly, or intrathecally, attenuated pain behaviors in the rodent models of inflammatory pain, visceral pain, and neuropathic pain [14, 18, 23, 24]. Taken together, all the results indicated that GPR40 agonists were specifically antinociceptive in painful hypersensitivity states.

The more striking finding from the current study is that GPR40 activation-induced antinociception is mediated by the spinal glial expression of IL-10 and subsequent β-endorphin. The notion is supported by the following findings: (1) Intrathecal injection of GW9508 stimulated the expression of IL-10 and β-endorphin in microglia and astrocytes, but not in neurons, in both contralateral and ipsilateral spinal cords of neuropathic rats, directly identified by double immunofluorescence staining of IL-10 and β-endorphin with cellular biomarkers, i.e., Iba-1, GFAP, and NeuN. (2) Treatment with GW9508 stimulated the gene and protein expression of IL-10 and β-endorphin in primary cultures of microglia and astrocytes, but not of neurons, in a GW1100-reversible manner. The specific IL-10 neutralizing antibody in primary microglia completely inhibited the GW9508-stimulated mRNA expression of POMC but not IL-10, whereas the β-endorphin antibody did not affect the GW9508-stimulated IL-10 or POMC gene expression. The results are in agreement with our previous findings that the IL-10 neutralizing antibody completely inhibited the exenatide-stimulated expression of POMC [39] and that IL-10 stimulated the β-endorphin expression but not vice versa [46]. (3) Intrathecal injection of the IL-10 neutralizing antibody, β-endorphin antiserum, and μ-opioid receptor-preferred antagonist naloxone entirely eliminated the spinal GW9508-induced mechanical antiallodynia in neuropathic rats. (4) Minocycline is generally deemed as a microglial inhibitor [53–55], but it also inhibited astrocyte activation as well [56–58]. Treatment with minocycline in the current study inhibited the GW9508-stimulated IL-10 and POMC gene expression in the cultured primary microglia and astrocytes. Moreover, the intrathecal injection of minocycline completely blocked the spinal GW9508-induced mechanical antiallodynia in neuropathic rats. These results further highlight the broad significance of the newly discovered spinal glial IL-10/β-endorphin pathway in pain process and modulation. It was recently discovered that IL-10 and IL-24, a member of the IL-10 family, were effective in relieving neuropathic pain and bone cancer pain in rats by the promotion of β-endorphin synthesis [62]. Moreover, GLP-1 receptor agonist exenatide induced the antinociception through spinal microglial IL-10 and β-endorphin pathway via the cAMP/PKA/p38β/CREB and JAK1/STAT3 signal transduction mechanisms [49].

β-Endorphin is an endogenous opioid neuropeptide that is known to be produced in neurons within the central nervous system including the hypothalamus and modulates pain transmission and transduction by the activation of μ-opioid receptors [63–67]. However, recent findings indicate that endogenous opioid peptides (including endorphins and dynorphins) are also present in astrocytes and microglia [29, 33, 39, 68, 69]. Particularly, β-endorphin and dynorphin A secreted by GLP-1 receptor agonists, herbal cynandione A, and aconitines effectively attenuated painful hypersensitivity [29–35]. Studies in the literature are controversial regarding the cell type specificity of the expression of β-endorphin and GPR40. GPR40 signaling was reported to enhance neuronal β-endorphin production in the hypothalamus in mice [28]. Moreover, GPR40 was reported to be specifically expressed on neurons in the mouse brain including POMC-positive neurons of the arcuate nucleus, serotonergic neurons in the nucleus raphe magnus, and noradrenergic neurons in the locus coeruleus, but rarely on astrocytes or microglia [14, 18, 25]. In contrast, our study demonstrated that GPR40 was expressed on rat microglia, astrocytes, and neurons in primary cultures (with 100% positive in each cell type) and in the spinal dorsal horns in vivo; the expression was upregulated by peripheral nerve injury. In addition, other laboratories also reported that GPR40 was expressed in astrocytes and neurons in the hippocampus of monkeys and the spinal cord of mice [4]. The reasons are not known for the discrepancy between the studies but may be related to the difference between animal species (rat, mouse vs. monkey), location of the central nervous system (brain vs. spinal cords), and even the selectivity of the antibodies used. Nevertheless, our systemic study, by double immunostaining of β-endorphin and GPR40, using combined in vivo spinal cords and cultured primary cells, measuring both the baseline and stimulated gene and protein expression, and employing the glial inhibitor, clearly illustrates the role of spinal glial IL-10/β-endorphin pathway in the GPR40 activation-induced antinociception.

GPR40 has been extensively reported to be involved in anti-inflammation. Topical administration of GW9508 had suppressed ear swelling and epidermal thickening and attenuated cell infiltration and contact hypersensitivity, with the downregulation of CCL10 and CXCL5 in either Th1- or Th2-type skin inflammation induced by DNFB [12]. A study conducted by Fujita et al. showed that GW9508 inhibited the expression of chemokines (CCL17, CCL10, and CCL5) and proinflammatory cytokines (IL-11, IL-24, and IL-33) in HaCaT and NHEK cells activated by TNF-α and IFN-γ [12]. Moreover, the GPR40 agonists could reduce inflammatory signaling, such as NF-κB, IL-1β, TNF-α, and NOS2α, in the mouse NIT1 insulinoma cells and rat islets that are under chronic inflammatory conditions [70] and LPS-induced activation of IKK in BV2 cells [71]. The underlying mechanism is not clear; however, the increased expression of IL-10 followed by GPR40 activation may be implicated in the GPR40-induced anti-inflammation, as IL-10 is recognized as an important anti-inflammatory cytokine [46]. Further researches are warranted to illustrate the causal association between the increased expression of IL-10 in macrophages and other cells and GPR40-induced anti-inflammation.

GPR40 and GLP-1 receptors are family A and B of G protein-coupled receptors, respectively. They share the same effects on insulin secretion and regulation of glucose metabolism in the periphery tissue and antinociception in the central nervous system. Particularly, the same IL-10/β-endorphin pathway is involved in both GLP-1 receptor and GPR40 activation-induced antinociception. However, GPR40 and GLP-1 receptor are associated with IL-10 expression through different phosphorylation mechanisms of MAPKs. Exenatide specifically activated p38 but not ERK or JNK MAPK. In addition, the exenatide-induced stimulation of IL-10 expression was inhibited by the specific p38 inhibitor, but not by the ERK or JNK inhibitors [39]. In contrast, GW9508 non-selectively activated p38, JNK, and ERK MAPK, and each specific MAPK inhibitor blocked the GW9508-induced IL-10 and subsequent POMC mRNA overexpression. The results suggest that GPR40 activation stimulates the IL-10 expression via cAMP/PKA/MAPK/CREB pathway and subsequently secretes β-endorphin though IL-10/IL-10 receptor/STAT3 pathway, the latter of which was demonstrated recently [46, 49].

## Conclusions

GW9508 given intrathecally produced mechanical antiallodynia and thermal antihyperalgesia in neuropathic rats and stimulated spinal glial expression of IL-10 and β-endorphin expression. The GW9508-induced mechanical antiallodynia was totally blocked by the specific GPR40 antagonist GW1100, glial inhibitor minocycline, IL-10 neutralizing antibody, β-endorphin antiserum, and μ-opioid receptor-preferred antagonist naloxone. In addition, the IL-10 neutralizing antibody inhibited the GW9508-stimulated mRNA expression of POMC but not IL-10, whereas the β-endorphin antibody did not affect the GW9508-stimulated IL-10 or POMC gene expression. Furthermore, treatment with GW9508 stimulated the phosphorylation of MAPKs and its stimulation of IL-10 expression was inhibited by each MAPK subtype inhibitor. Our results reveal that GPR40 activation-induced antinociception in neuropathic pain is through the spinal glial expression of IL-10 followed by β-endorphin secretion, and highlight the broad significance of the glial IL-10/β-endorphin pathway in the regulation of antinociception.

## Additional files

Additional file 1: **Figure S1.** Expression of GPR40 in the spinal dorsal horn of neuropathic rats induced by L5/L6 spinal nerve ligation. Frozen sections were obtained from spinal lumbar enlargements from neuropathic rats approximately 2 weeks after surgery. Immunofluorescence was stained with the GPR40 antibody and photomicrographs were taken from the entire spinal cord section (**A**, 500 μm). **B**. The immunolabeled surface areas of GPR40 from the spinal dorsal horn laminae I-V indicated in white lines were quantified using the ImageJ program. Data are presented as mean ± SEM (*N* = 11~12 per group). * *P* < 0.05, vs saline group; analyzed by unpaired and two-tailed Student t-test. (ZIP 300 kb)

Additional file 2: **Figure S2.** Stimulatory effect of intrathecal injection of GW9508 on IL-10 expression in the spinal dorsal horn of neuropathic rats induced by L5/L6 spinal nerve ligation. The spinal lumbar enlargements were obtained 1 hour after intrathecal injection of saline (10 μl) or GW9508 (30 μg). For the gene and protein analysis study, expression of the IL-10 gene (**A**) and protein (**B**) levels were determined using qRT-PCR and a specific fluorescent immunoassay kit, respectively. For the immunostaining study, the spinal lumbar enlargements were frozen. Immunofluorescence was stained with the IL-10 antibody and photomicrographs were taken from the entire spinal cord section. (**C**, **D**, 500 μm). **E**. The immunolabeled surface areas of IL-10 from the spinal dorsal horn laminae I-V indicated in white lines were quantified using the ImageJ program. Data are presented as mean ± SEM (*N* = 5~6 per group). * *P* < 0.05, vs saline group; analyzed by unpaired and two-tailed Student t-test. (ZIP 447 kb)

Additional file 3: **Figure S3.** Stimulatory effect of intrathecal injection of GW9508 on β-endorphin expression in the spinal dorsal horn of neuropathic rats induced by L5/L6 spinal nerve ligation. The spinal lumbar enlargements were obtained 1 hour after intrathecal injection of saline (10 μl) or GW9508 (30 μg). For the gene and protein analysis study, expression of the β-endorphin precursor POMC gene (**A**) and β-endorphin protein (**B**) levels were determined using qRT-PCR and a specific fluorescent immunoassay kit, respectively. For the immunostaining study, the spinal lumbar enlargements were frozen. Immunofluorescence was stained with the β-endorphin antibody and photomicrographs were taken from the entire spinal cord section (**C**, **D**, 500 μm). **E**. The immunolabeled surface areas of β-endorphin from the spinal dorsal horn laminae I-V indicated in white lines were quantified using the ImageJ program. Data are presented as mean ± SEM (*N* = 5~8 per group). * *P* < 0.05, vs saline group; analyzed by unpaired and two-tailed Student t-test. (ZIP 464 kb)

## Abbreviations

CFA: Complete Freund’s adjuvant
DHA: Docosahexaenoic acid
DMSO: Dimethyl sulfoxide
ERK: Extracellular signal-regulated kinase
FFA: Free fatty acid
GFAP: Glial fibrillary acid protein
GLP-1: Glucagon-like peptide-1
GPR40: G protein-coupled receptor 40
Iba-1: Ionized calcium binding adaptor molecule-1
IL-10: Interleukin-10
JNK: c-Jun N-terminal kinase
MAPK: Mitogen-activated protein kinase
MPE: Maximal possible effect
NeuN: Neuronal specific nuclear protein
PBS: Phosphate-buffered saline
PDYN: Prodynorphin
PEG: Polyethylene glycol
POMC: Proopiomelanocortin
RIPA: Radioimmunoprecipitation assay
SDS-PAGE: Sodium dodecyl sulfatepolyacrylamide gel electrophoresis
