# Supplementary material for: Activation of GPR40 produces mechanical antiallodynia via the spinal glial interleukin-10/β-endorphin pathway
Source: J Neuroinflammation. 2019 Apr 13;16:84. doi: 10.1186/s12974-019-1457-9 (PMC6461825; doi:10.1186/s12974-019-1457-9)
Supplement: Supplementary file 1 — Figure S1. Expression of GPR40 in the spinal dorsal horn of neuropathic rats induced by L5/L6 spinal nerve ligation. Frozen sections were obtained from spinal lumbar enlargements from neuropathic rats approximately 2 weeks after surgery. Immunofluorescence was stained with the GPR40 antibody and photomicrographs were taken from the entire spinal cord section (A, 500 μm). B. The immunolabeled surface areas of GPR40 from the spinal dorsal horn laminae I-V indicated in white lines were quantified using the ImageJ program. Data are presented as mean ± SEM (N = 11~12 per group). * P < 0.05, vs saline group; analyzed by unpaired and two-tailed Student t-test. (ZIP 300 kb) [file 12974_2019_1457_MOESM1_ESM.zip › Fig.S1 legend.docx]

**Fig. S1** Expression of GPR40 in the spinal dorsal horn of neuropathic rats induced by L5/L6 spinal nerve ligation. Frozen sections were obtained from spinal lumbar enlargements from neuropathic rats approximately 2 weeks after surgery. Immunofluorescence was stained with the GPR40 antibody and photomicrographs were taken from the entire spinal cord section (**A,** 500 μm). **B**. The immunolabeled surface areas of GPR40 from the spinal dorsal horn laminae I-V indicated in white lines were quantified using the ImageJ program. Data are presented as mean ± SEM (N=11~12 per group). * P<0.05, vs saline group; analyzed by unpaired and two-tailed Student t-test.
