# Supplementary figures and images for: Activation of GPR40 produces mechanical antiallodynia via the spinal glial interleukin-10/β-endorphin pathway
Source: J Neuroinflammation. 2019 Apr 13;16:84. doi: 10.1186/s12974-019-1457-9 (PMC6461825; doi:10.1186/s12974-019-1457-9)

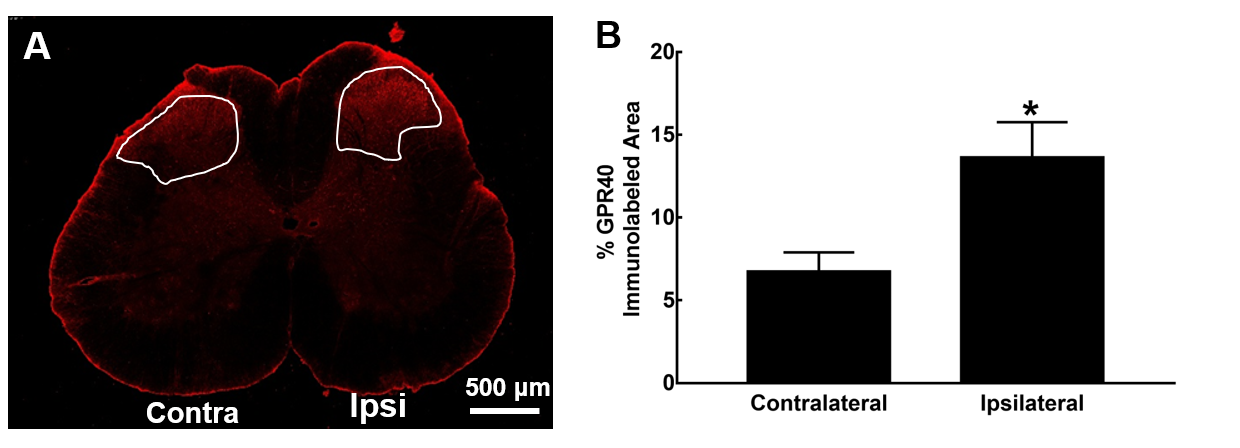

Supplement: Supplementary file 1 — Figure S1. Expression of GPR40 in the spinal dorsal horn of neuropathic rats induced by L5/L6 spinal nerve ligation. Frozen sections were obtained from spinal lumbar enlargements from neuropathic rats approximately 2 weeks after surgery. Immunofluorescence was stained with the GPR40 antibody and photomicrographs were taken from the entire spinal cord section (A, 500 μm). B. The immunolabeled surface areas of GPR40 from the spinal dorsal horn laminae I-V indicated in white lines were quantified using the ImageJ program. Data are presented as mean ± SEM (N = 11~12 per group). * P < 0.05, vs saline group; analyzed by unpaired and two-tailed Student t-test. (ZIP 300 kb) [file 12974_2019_1457_MOESM1_ESM.zip › Fig.S1.tif]

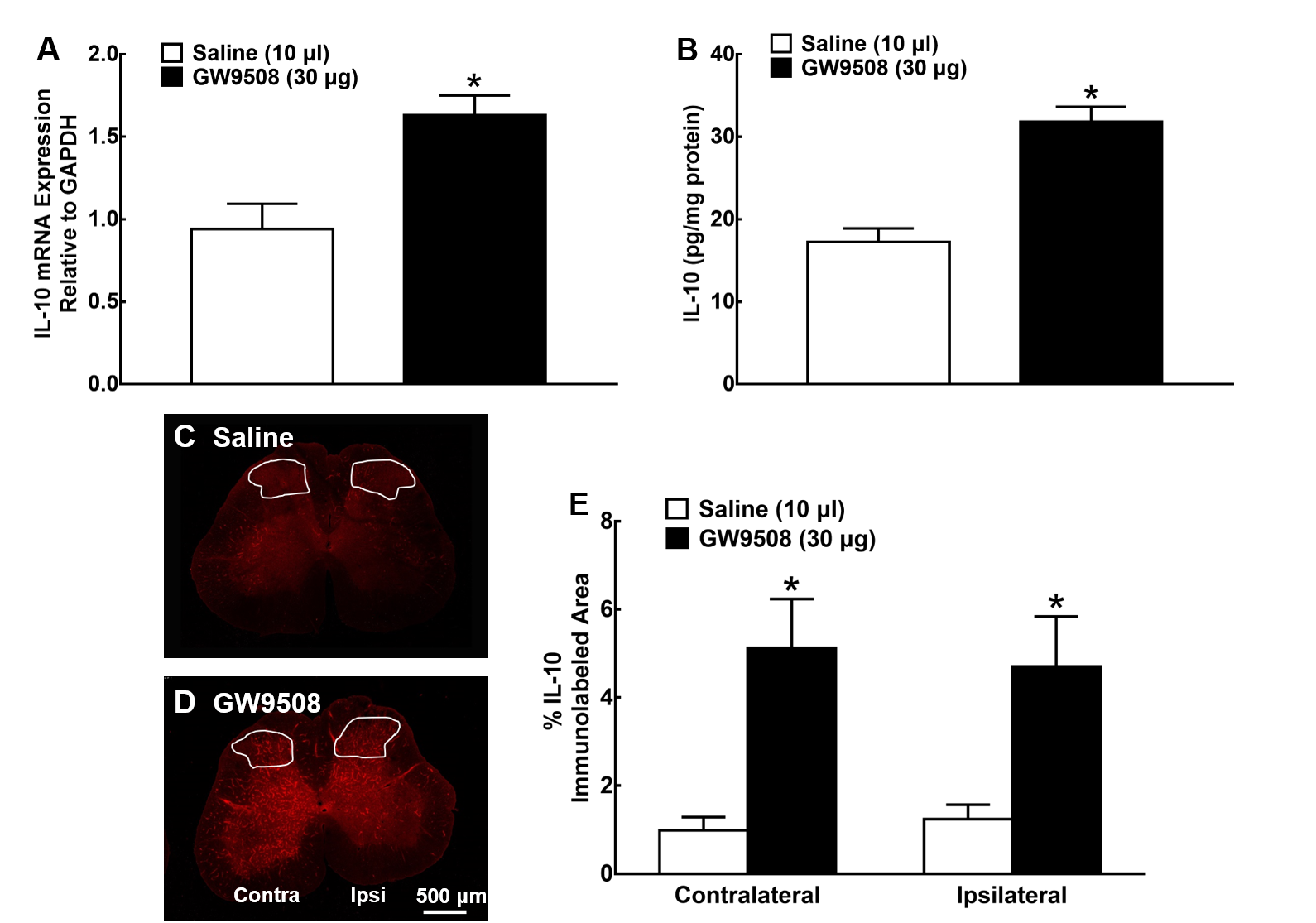

Supplement: Supplementary file 2 — Figure S2. Stimulatory effect of intrathecal injection of GW9508 on IL-10 expression in the spinal dorsal horn of neuropathic rats induced by L5/L6 spinal nerve ligation. The spinal lumbar enlargements were obtained 1 hour after intrathecal injection of saline (10 μl) or GW9508 (30 μg). For the gene and protein analysis study, expression of the IL-10 gene (A) and protein (B) levels were determined using qRT-PCR and a specific fluorescent immunoassay kit, respectively. For the immunostaining study, the spinal lumbar enlargements were frozen. Immunofluorescence was stained with the IL-10 antibody and photomicrographs were taken from the entire spinal cord section. (C, D, 500 μm). E. The immunolabeled surface areas of IL-10 from the spinal dorsal horn laminae I-V indicated in white lines were quantified using the ImageJ program. Data are presented as mean ± SEM (N = 5~6 per group). * P < 0.05, vs saline group; analyzed by unpaired and two-tailed Student t-test. (ZIP 447 kb) [file 12974_2019_1457_MOESM2_ESM.zip › Fig.S2.tif]

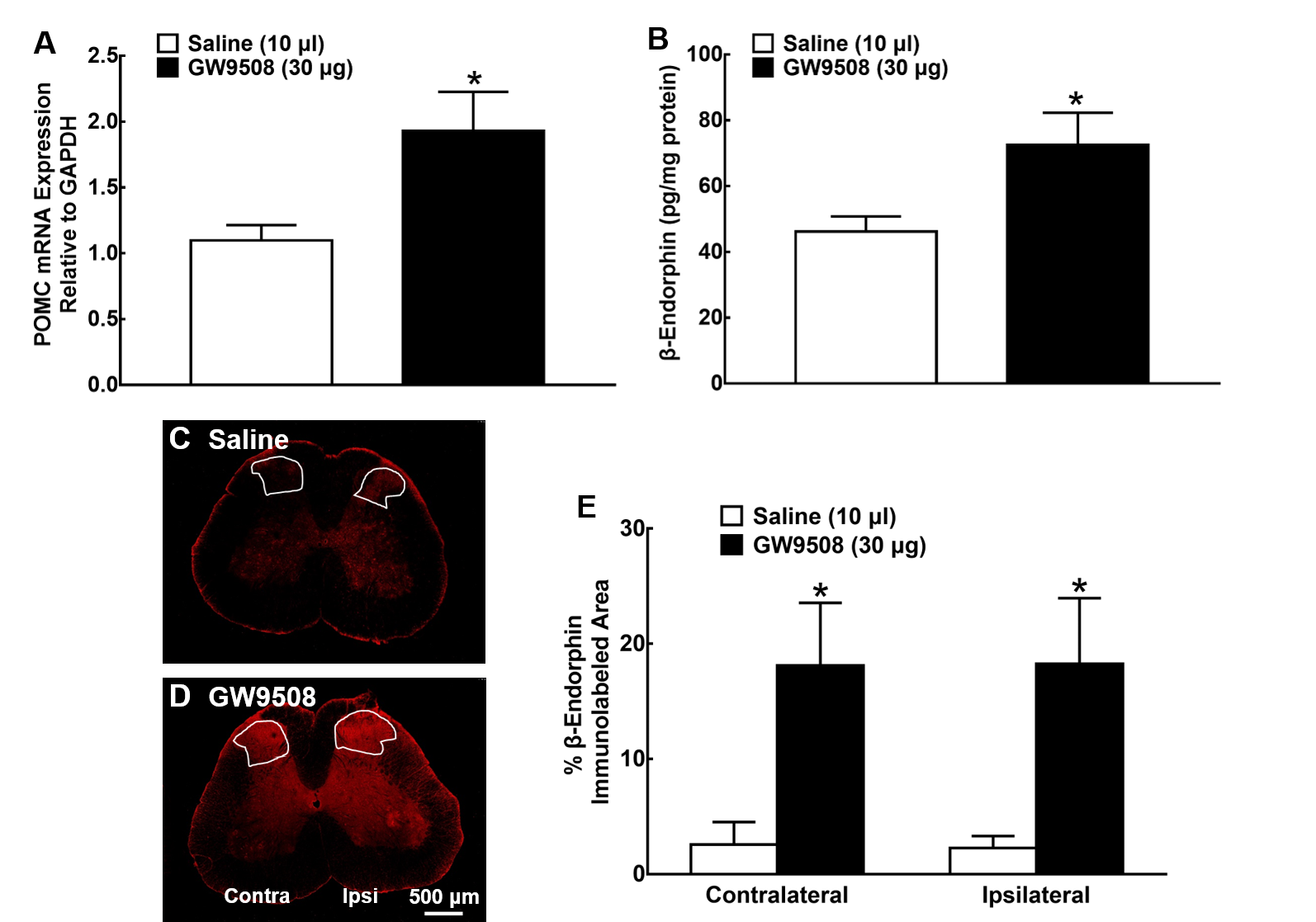

Supplement: Supplementary file 3 — Figure S3. Stimulatory effect of intrathecal injection of GW9508 on β-endorphin expression in the spinal dorsal horn of neuropathic rats induced by L5/L6 spinal nerve ligation. The spinal lumbar enlargements were obtained 1 hour after intrathecal injection of saline (10 μl) or GW9508 (30 μg). For the gene and protein analysis study, expression of the β-endorphin precursor POMC gene (A) and β-endorphin protein (B) levels were determined using qRT-PCR and a specific fluorescent immunoassay kit, respectively. For the immunostaining study, the spinal lumbar enlargements were frozen. Immunofluorescence was stained with the β-endorphin antibody and photomicrographs were taken from the entire spinal cord section (C, D, 500 μm). E. The immunolabeled surface areas of β-endorphin from the spinal dorsal horn laminae I-V indicated in white lines were quantified using the ImageJ program. Data are presented as mean ± SEM (N = 5~8 per group). * P < 0.05, vs saline group; analyzed by unpaired and two-tailed Student t-test. (ZIP 464 kb) [file 12974_2019_1457_MOESM3_ESM.zip › Fig.S3.tif]
