# Supplementary material for: Activation of GPR40 produces mechanical antiallodynia via the spinal glial interleukin-10/β-endorphin pathway
Source: J Neuroinflammation. 2019 Apr 13;16:84. doi: 10.1186/s12974-019-1457-9 (PMC6461825; doi:10.1186/s12974-019-1457-9)
Supplement: Supplementary file 2 — Figure S2. Stimulatory effect of intrathecal injection of GW9508 on IL-10 expression in the spinal dorsal horn of neuropathic rats induced by L5/L6 spinal nerve ligation. The spinal lumbar enlargements were obtained 1 hour after intrathecal injection of saline (10 μl) or GW9508 (30 μg). For the gene and protein analysis study, expression of the IL-10 gene (A) and protein (B) levels were determined using qRT-PCR and a specific fluorescent immunoassay kit, respectively. For the immunostaining study, the spinal lumbar enlargements were frozen. Immunofluorescence was stained with the IL-10 antibody and photomicrographs were taken from the entire spinal cord section. (C, D, 500 μm). E. The immunolabeled surface areas of IL-10 from the spinal dorsal horn laminae I-V indicated in white lines were quantified using the ImageJ program. Data are presented as mean ± SEM (N = 5~6 per group). * P < 0.05, vs saline group; analyzed by unpaired and two-tailed Student t-test. (ZIP 447 kb) [file 12974_2019_1457_MOESM2_ESM.zip › Fig.S2 legend.docx]

**Fig. S2** Stimulatory effect of intrathecal injection of GW9508 on IL-10 expression in the spinal dorsal horn of neuropathic rats induced by L5/L6 spinal nerve ligation. The spinal lumbar enlargements were obtained 1 hour after intrathecal injection of saline (10 μl) or GW9508 (30 μg). For the gene and protein analysis study, expression of the IL-10 gene (**A**) and protein (**B**) levels were determined using qRT-PCR and a speciﬁc ﬂuorescent immunoassay kit, respectively. For the immunostaining study, the spinal lumbar enlargements were frozen. Immunofluorescence was stained with the IL-10 antibody and photomicrographs were taken from the entire spinal cord section. (**C, D,** 500 μm). **E**. The immunolabeled surface areas of IL-10 from the spinal dorsal horn laminae I-V indicated in white lines were quantified using the ImageJ program. Data are presented as mean ± SEM (N=5~6 per group). * P<0.05, vs saline group; analyzed by unpaired and two-tailed Student t-test.
